# Rapid Evolution of HERC6 and Duplication of a Chimeric HERC5/6 Gene in Rodents and Bats Suggest an Overlooked Role of HERCs in Mammalian Immunity

**DOI:** 10.3389/fimmu.2020.605270

**Published:** 2020-12-18

**Authors:** Stéphanie Jacquet, Dominique Pontier, Lucie Etienne

**Affiliations:** ^1^ Université de Lyon, Université Lyon 1, CNRS, Laboratoire de Biométrie et Biologie Évolutive UMR 5558, Villeurbanne, France; ^2^ CIRI—Centre International de Recherche en Infectiologie, Univ Lyon, Inserm U1111, Université Claude Bernard Lyon 1, CNRS UMR5308, ENS de Lyon, Lyon, France; ^3^ LabEx Ecofect, Université de Lyon, Lyon, France

**Keywords:** HERC, restriction factor, antiviral immunity, gene duplication, genetic conflicts, positive selection, HERC5, HERC6

## Abstract

Studying the evolutionary diversification of mammalian antiviral defenses is of main importance to better understand our innate immune repertoire. The small HERC proteins are part of a multigene family, including HERC5 and HERC6, which have probably diversified through complex evolutionary history in mammals. Here, we performed mammalian-wide phylogenetic and genomic analyses of HERC5 and HERC6, using 83 orthologous sequences from bats, rodents, primates, artiodactyls, and carnivores—the top five representative groups of mammalian evolution. We found that HERC5 has been under weak and differential positive selection in mammals, with only primate HERC5 showing evidences of pathogen-driven selection. In contrast, HERC6 has been under strong and recurrent adaptive evolution in mammals, suggesting past and widespread genetic arms-races with viral pathogens. Importantly, the rapid evolution of mammalian HERC6 spacer domain suggests that it might be a host-pathogen interface, targeting viral proteins and/or being the target of virus antagonists. Finally, we identified a HERC5/6 chimeric gene that arose from independent duplication in rodent and bat lineages and encodes for a conserved HERC5 N-terminal domain and divergent HERC6 spacer and HECT domains. This duplicated chimeric gene highlights adaptations that potentially contribute to rodent and bat immunity. Our findings open new research avenues on the functions of HERC6 and HERC5/6 in mammals, and on their implication in antiviral innate immunity.

## Introduction

As a result of sustained exposure to viral infections, mammals have evolved a sophisticated and diversified immune repertoire against viruses. A hallmark of mammalian antiviral immunity is the induction of type I interferon (IFN) upon viral infection. This cytokine upregulates the transcription of hundreds of interferon-stimulated genes (ISGs) in viral infected cells (1). Many of these ISGs encode proteins with antiviral functions, named restriction factors, which are critical players in the first line of the innate immune defense inhibiting different steps of the viral replication cycle (2).

Viruses have adapted to circumvent, subvert, or antagonize these host restriction factors (3). Reciprocally, restriction factors have rapidly and repeatedly evolved to maintain defenses against evolving viral pathogens, leading to virus-host evolutionary arms-races (3, 4). These dynamics of reciprocal adaptations can leave genetic signatures in the host restriction factors. Significant accumulations of non-synonymous changes over synonymous substitutions—designated as positive selection, as well as codon deletions or insertions that may alter the virus-host interface—are common genetic signatures of such long-term evolutionary arms-races (3–6). At the genomic level, gene duplication and recombination are among the most important mechanisms that can diversify the antiviral immune repertoire. In particular, gene duplication can generate adaptive molecular novelty allowing hosts to escape viral antagonism, evolve new immune functions, or increase the depth of antiviral response (3, 7, 8). The weight of such evolutionary mechanisms in mammalian immunity is highlighted by the extent of multigene families, which encode important ISG-encoded proteins, such as the Tripartite Motif-containing (TRIM) (9–12), Apolipoprotein B Editing Complex (APOBEC3) (13–15), Interferon-induced Protein with Tetratricopeptide Repeats (IFIT) (16–18), Interferon induced Transmembrane protein (IFITM) (19) families. For example, the *APOBEC*3 family has expanded in a lineage-specific manner in primates (20), artiodactyls (21), and bats (15), generating variability in mammalian antiviral response (14). However, the evolutionary and functional diversification of many antiviral families remains poorly characterized in mammals. Deciphering the evolutionary trajectories of multigene family members can provide insights into the genetic mechanisms underlying the diversification of antiviral responses and may allow identifying novel antiviral proteins.

The HECT and RLD domain containing E3-ubiquitin protein ligases, known as HERC proteins, are encoded by a multigene superfamily that is poorly studied in mammals. With six gene members, the HERC family is divided into two subfamilies, the large (*HERC1* and *HERC2*) and the small (*HERC3–6*) *HERCs* (22). The small HERC proteins are structurally characterized by a N-terminal RCC1-like domain (RLD), a spacer region, and a C-terminal HECT (Homologous E6-AP Carboxyl Terminus) ubiquitin E3-ligase domain, while the large HERCs possess at least two RLD domains in addition to a HECT domain (23). This structural difference between large and small HERCs reflects their independent evolutionary history (24). In the antiviral immune context, much attention has been devoted to the small HERCs, in particular to HERC5 - an ISG-encoded antiviral effector - and HERC6 its closest relative (24, 25). In humans, HERC5 acts as a HECT ubiquitin and E3-ligase (26–28). HERC5 notably conjugates the ubiquitin-like protein ISG15 to different protein targets, a process termed ISGylation (29–31). The protein targets may be non-specific newly synthetized viral proteins, specific viral proteins, or specific host proteins (29–31). ISGylated proteins are modified, functionally disrupted, or altered in their localization within the cells. Through this ISGylation activity, HERC5 has an antiviral function against highly divergent viruses, including retrovirus (Human and Simian immunodeficiency viruses, HIV and SIV), papillomavirus, and influenza virus (25, 31–33). For example, HERC5 targets the early stage of HIV assembly by catalyzing the ISGylation of the viral Gag protein (30), while it reduces influenza A viral replication through the conjugation of ISG15 to the viral NS1 protein (31). Besides, HERC5 appears to further interfere with HIV replication in an ISGylation-independent manner by impacting the nuclear export of Rev/RRE-dependent viral RNA, most likely through determinants in the RLD domain (33). In contrast, although HERC6 is the most closely-related protein of HERC5, little is known about its functional implication in mammalian antiviral immunity (25). The antiviral role of HERC6 has mainly been described in mouse, in which the *HERC5* gene has been lost and functionally substituted by HERC6, the main murine E3-ligase of ISG15 (28, 34, 35). In humans, although the HERC6 protein possesses a HECT E3-ligase domain, it is devoid of ISGylation function (25).

These evolutionary and functional differences between mammals suggest lineage-specific adaptive changes in *HERC5* and *HERC6*. Two previous studies showed that *HERC5* and *HERC6* genes have evolved under positive selection during vertebrate evolution (25, 33). They further showed that the RLD domain plays an important role in the antiviral activity of HERC5 and HERC6 proteins. While these studies have provided important insights into the diversification of *HERCs*, the evolutionary analyses have certain limitations: (i) the scarcity of species analyzed (only 12 species, *versus* 81 species with at least 10 species per order in this current study), (ii) the overrepresentation of primates compared to other mammalian species (seven primates, two to three carnivores, two artiodactyls, and one perissodactyl), (iii) the integration of highly divergent species, which may bias the genetic inferences by increasing the number of false positives (36). Moreover, a recent study in primates have shown differences in *HERC5* and *HERC6* selective pressures (37). Therefore, how *HERC5* and HERC6 genes have evolved within mammalian orders has not been fully deciphered. Nor is the evolutionary dynamic of *HERC5* and *HERC6* expansions and contractions across mammals.

Here, we decipher the evolutionary history of mammalian *HERC5* and *HERC6 via* mammalian-wide and lineage-specific phylogenetic and genomic analyses. We analyzed the orthologous sequences of *HERC5* and *HERC6* from bats, rodents, primates, artiodactyls, and carnivores the top five mammalian orders in terms of zoonotic viral diversity they host (38, 39). First, we show that *HERC6*—and to a much lesser extent *HERC5*—has been under strong positive selection. Second, we stressed the HERC6 spacer region as a potential pathogen-mammal interface, targeting viral proteins and/or being the target of virus antagonists. Finally, we identified independent gene duplications through recombination between *HERC5* and *HERC6* in some bat and rodent lineages, which have led to the fixation of a chimeric *HERC5/6* gene in both mammalian orders. Taken together, our results suggest that HERC6 may be an important antiviral protein in mammals and identified a novel chimeric HERC member in bats and rodents that may contribute to unique antiviral responses in these species.

## Materials and Methods

### Collection of Mammalian *HERC5* and *HERC6* Orthologous Sequences

Full-length *HERC5* and *HERC6* coding sequences were analyzed in bats, rodents, primates, artiodactyls, and carnivores. *HERC5* and *HERC6* coding sequences from each group were obtained using the Little Brown bat (*Myotis lucifigus*), mouse (*Mus musculus*), human (*Homo sapiens*), cow (*Bos taurus*), and dog (*Canis lupus familiaris*) Refseq proteins as queries, respectively, through tBLASTn searches of the “Nucleotide” database in GenBank (40, 41). The species and accession numbers are presented in **Supplementary Table 1**.

### Characterizing the Evolution of *HERC5* and *HERC6* Synteny in Mammals

The genomic locus of *HERC5* and *HERC6* genes in Little Brown bat, mouse, human, dog, and cow were obtained from Ensembl (http://www.ensembl.org/index.html), and GenBank Refseq genome database (41). Their coding sequences were used as queries for BLASTn searches against a total of 110 whole genome assemblies from bats, rodents, primates, carnivores, and artiodactyls (**Supplementary Table 1**) available in GenBank database (41). We analyzed eight additional mammalian genomes from Proboscidea, Lagomorpha, Scandentia, Perissodactyla, Sirenia, Soricomorpha, Eulipotyphla, and Tubulidentata orders (*Orycteropus afer*, *Loxodonta Africana*, *Trichechus manatus*, *Tupaia chinensis*, *Condylura cristata*, *Ceratotherium simum*, *Equus przewalskii*, and *Sorex araneus*, respectively) (**Supplementary Table 1**). The synteny of *HERC5* and *HERC6* genes was analyzed and visualized through BLAST searches against the 110 annotated genomes in GenBank database (41). Newly identified HERC-like paralogs (see *Results*) were confirmed by blasting and aligning their whole sequence (intron and exon regions) to the genomic region containing *HERC5* and *HERC6* genes in three bat species (*Myotis lucifigus*, *Rhinolophus ferrumequinum*, and *Phyllostomus discolor*), and three rodent species (*Chinchilla lanigera*, *Mastomys coucha*, *Cavia porcellus*), which are representative genomes with good assembling quality (based on the N50, the number of scaffolds, and sequencing coverage).

*HERC5* and *HERC6* orthologs as well as *HERC-like* paralogous sequences were aligned for each mammalian order separately using the program MACSE (42), and the alignments were manually curated. A phylogenetic tree was then built for each gene and mammalian order, and for a combined dataset of *HERC5* and *HERC6* genes (rooted with *HERC3* as an outgroup, which is the most closely related gene to *HERC5* and *HERC6*), using the maximum likelihood method implemented in the ATGC-PhyML Web server (43). Each phylogenetic tree was based on the best substitution model (GTR+G+I), as determined by the Smart Model Selection (SMS) program in PhyML (44) and node statistical support was computed through 1,000 bootstrap replicates.

### Assessing Recombination Events in *HERC5* and *HERC6* Paralogs and Orthologs

To test whether recombination has occurred in *HERC5*, *HERC6*, and *HERC-like* genes, we ran the GARD (Genetic Algorithm for Recombination Detection) method (45) implemented in the HyPhy package (46, 47), using a general discrete site-to-site rate variation with three rate classes. The program uses a genetic algorithm to screen multiple-sequence alignment for putative recombination breakpoints and provides the probability of support for each breakpoint. GARD analyses were run for each mammalian order and each gene separately, including the newly identified HERC-like paralog.

### Positive Selection Analyses of *HERC5* and *HERC6* Coding Sequences in Mammals

To determine whether *HERC5* and *HERC6* have been targets of natural selection during mammalian evolution, we carried out positive selection analyses on orthologous coding sequences from bats (n = 10 and 13, respectively), rodents (n = 11 and 16), primates (n = 19 and 20), carnivores (n = 20 and 23), and artiodactyls (n = 21 and 11). As combining highly divergent sequences for positive selection analyses can lead to misleading results, we performed positive selection analyses on separate dataset for each mammalian order. For Artiodactyls, three different datasets were analyzed: the first dataset included all the available species, the second was restricted to cetacean species, and the third excluded the cetaceans. Analyzing each mammalian order separately allowed us to qualitatively compare the evolutionary profile of both genes in each mammalian order. We first tested for positive selection at the gene level using two different methods available in the Codeml program, which is implemented in the PAML package (48). This program allows both gene- and site-specific detection of positive selection by comparing constrained models that disallow positive selection (models M1 and M7) to unconstrained models allowing for positive selection (M2 and M8). We ran the different models with the codon frequencies of F61 and F3x4 with a starting omega ω (*dN*/*dS* ratio) of 0.4. Likelihood ratio tests were computed to compare the models (M1 *vs* M2 and M7 *vs* M8), and codons evolving under significant positive selection (*dN*/*dS >*1) were identified using the Bayes Empirical Bayes (BEB) with a posterior probability ≥0.95. The residues under positive selection were further assessed using two other methods, the Fast, Unconstrained Bayesian AppRoximation for Inferring Selection (FUBAR) (49) and the Mixed Effects Model of Evolution (MEME) (50), both implemented in the HYPHY package. To increase the specificity of our results, we only kept the sites that were identified as being under significant positive selection by at least two of the four methods used. When significant recombination breakpoints were detected, positive selection analyses were carried out for each fragment identified by GARD. Similarly, we sought for signatures of adaptive selection in the *HERC*-like paralogs of rodents (see *Results*), for which five orthologous coding sequences were available. Furthermore, we tested if the three HERC domains (RLD, spacer region, and HECT) have similarly been subjects of positive selection, by analyzing each domain separately.

Finally, to determine if *HERC5* and *HERC6* have experienced episodic selection within mammalian orders, we carried out the branch-specific analysis aBSREL (51, 52), implemented in the HYPHY package. This program allows testing the significance of positive selection and quantifying the *dN*/*dS* ratio for each branch independently.

## Results

### Lineage-Specific Changes in *HERC5* and *HERC6* Copy Number

**Figure 1:**
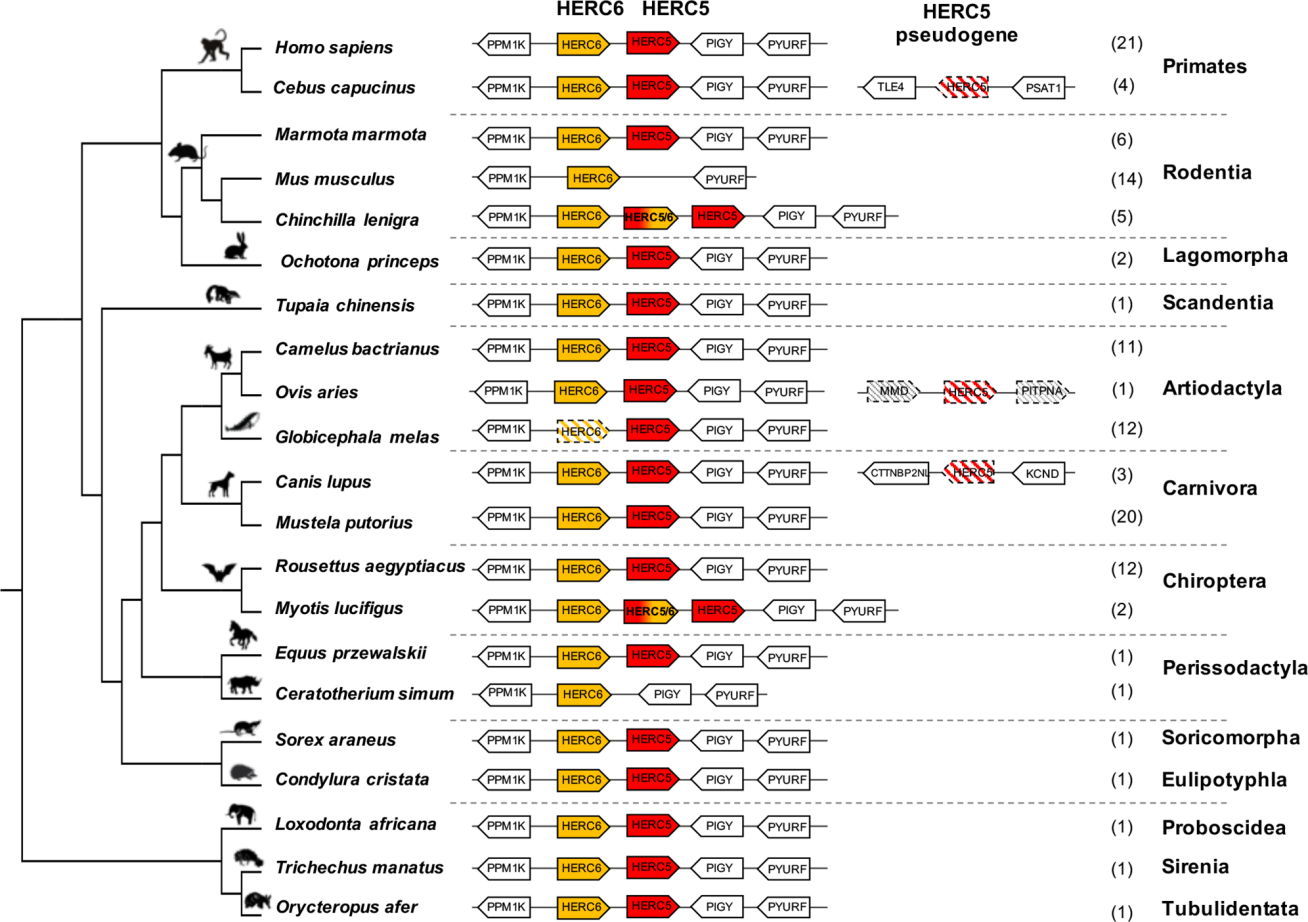
Evolutionary dynamics of mammalian *HERC5* and *HERC6* gene loci. Representation of the *HERC5* and *HERC6* gene loci from mammalian genomes. Plain colored arrows represent intact *HERC5* and *HERC6* genes, striped colored arrows indicate *HERC5* or *HERC6* pseudogenes, and white arrows are adjacent syntenic genes. The newly identified chimeric *HERC5/6* genes are bicolored. The numbers in brackets indicate the total number of genomes analyzed which contain the corresponding genomic organization in each mammalian order. In primates and carnivores, the *HERC5* and *HERC6* genes are well conserved. In the cetacean and rhinoceros’ species, the *HERC6* has been pseudogenized and lost, respectively, while rodent *HERC5* has been lost in the Muridae and Cricetidae families. A duplicated *HERC5/6* fused gene is independently fixed in several rodent species and in the *Myotis* genus in bats. Duplication followed by pseudogenization of *HERC5* in primates, carnivores and artiodactyls is presented.

To determine the genomic evolutionary history of *HERC5* and *HERC6* in mammals, we performed a complete synteny analysis for 14 chiropteran, 25 rodent, 25 primate, 23 artiodactyl, and 23 carnivore species, and eight more species from different orders. We first found that *HERC5* and *HERC6* synteny is mostly conserved throughout eutherian mammals (**Figure 1**, **Supplementary Figure 1**).

However, we also detected that gene erosion has repeatedly shaped the evolution of *HERC5* and *HERC6* in mammals. While primate and carnivore genomes encode both genes, the artiodactyls and the rodents have experienced gene loss or pseudogenization, generating *HERC* gene copy number variation in each group. Indeed, although all the studied artiodactyl species encode an intact *HERC5* gene, the cetacean genomes showed *HERC6* pseudogenization, through nucleotide deletions that impacted frameshift, as well as early stop codons (**Figure 1**, **Supplementary Figure 2**). Moreover, we confirmed the erosion of *HERC5* in rodents (25), and we further showed that this loss has most probably occurred in the common ancestor of the Cricetidae and Muridae. Interestingly, we also did not find the *HERC5* gene in the rhinoceros genome, suggesting at least two independent losses of *HERC5* in mammals, specifically in the Rodent and Perissodactyl orders (**Figure 1**).

Finally, in addition to these independent losses of *HERC5* or *HERC6* in mammals, we found multiple evidences of *HERC5* duplication followed by pseudogenization in primate (*Sapajus apella*, *Cebus capucinus*, *Aotus nancymaae*, and *Saimiri boliviensis*), carnivore (*Canis lupus*, *Vulpes Vulpes*, *leptonychotes weddellii*), and artiodactyl (*Ovis aries*) species, highlighting a strong dynamic of gene gain and loss in mammalian *HERC5* and *HERC6*.

### Ancient and Recent Recombinations Have Shaped the Evolution of a Duplicated Chimeric *HERC5/6* Gene in Rodents and Bats

While most mammalian species possess one or both *HERC* genes, we found independent duplications of *HERC5* in the chiropteran *Myotis* genus and the rodent Hystricognathi infra-order (**Figure 1**, **Supplementary Figure 3**). These duplicated genes were identified in three bat *Myotis* species (*M. lucifigus*, *M. brandtii*, and *M. davidii*) and five rodent species (*Fukomys damarensis*, *Heterocephalus glaber*, *Cavia porcellus*, *Chinchilla lanigera*, and *Octodon degus*) (**Table S1**). This dates these duplication events to at least 30 MYA (million years ago) and 44 MYA, respectively.

**Figure 2:**
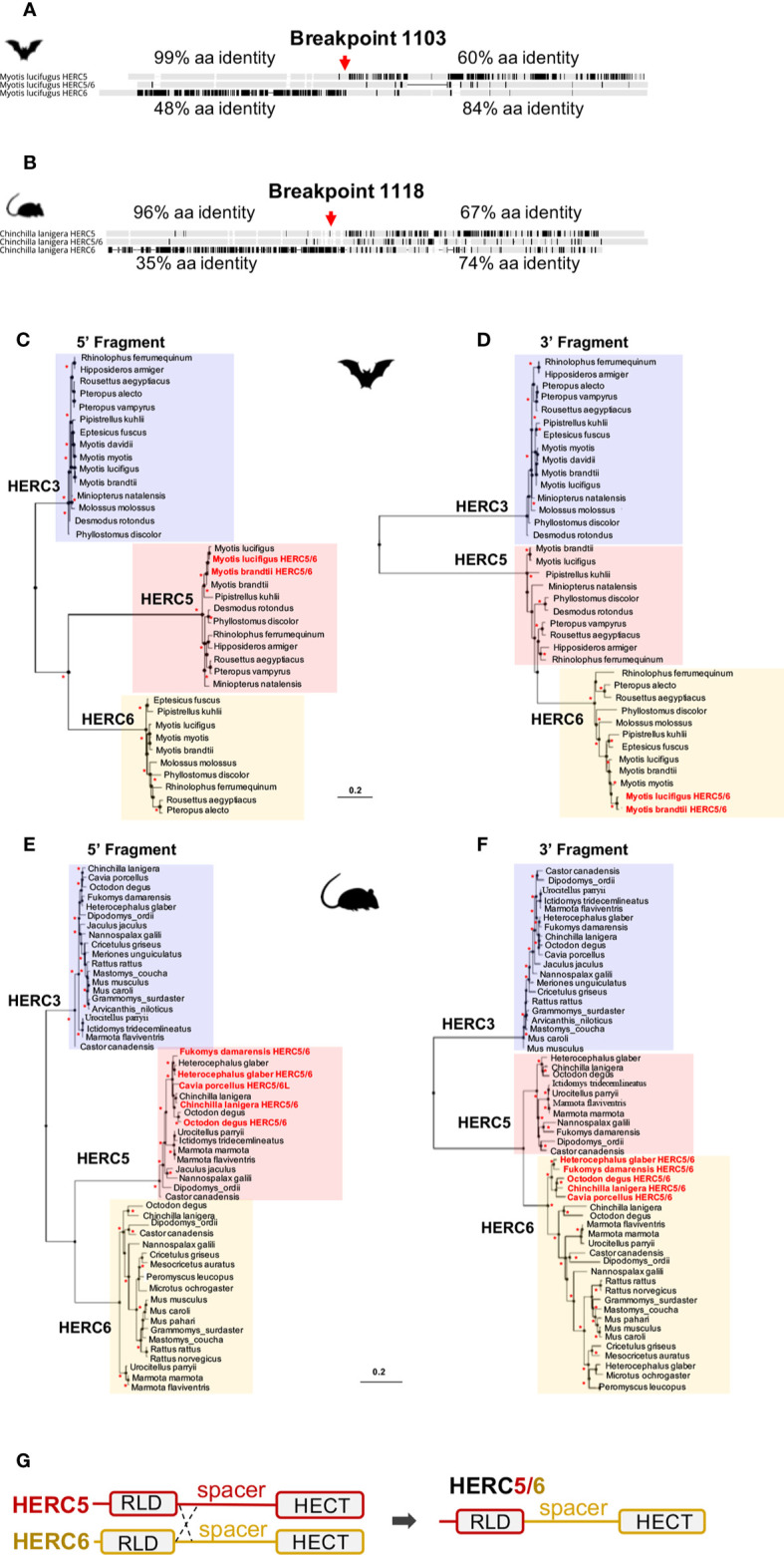
Independent duplication of a chimeric *HERC5/6* gene through recombination in bats and rodents **(A, B)**. Alignment of the protein sequence of HERC5, HERC5/6, and HERC6 genes from bats and rodents, respectively. Additional sequence alignments are shown in Supplementary Figure 4. The percentages of pairwise amino acid identities between the N-terminals of HERC5/6 and HERC5 or HERC6, as well as the C-terminals of HERC5/6 and HERC5 or HERC6 are indicated. The significant recombination breakpoints (red arrows, p-value <0.05) assigned by the GARD program are shown for the rodent and bat HERC5/6 gene **(C–F)**. Maximum likelihood phylogenetic tree generated with the 5’ (at the left) and 3’ (at the right) of the *HERC5*, *HERC6*, *HERC5/6*, and *HERC3* (as an outgroup) nucleotide gene alignment based on GARD recombination results (corresponding to the breakpoint 1103 in *Myotis lucifigus* in bats, and 1118 in *Chinchilla lanigera* in rodents). The duplicated chimeric *HERC5/6* genes are shown in red. Asterisks indicate bootstrap values greater than 80%. The scale bar represents genetic variation of a 0.2 (20%) for the length of the scale **(G)**. A linear representation of *HERC5* and *HERC6* structures showing a chromosomal crossover between the 5’ regions, upstream of the spacer region. This mechanism has led to a duplicated recombined *HERC5/6* gene containing the HERC5 RLD domain and HERC6 spacer region and HECT domain.

Surprisingly, alignments of HERC5, HERC-like, and *HERC6* proteins in each mammalian group revealed 96–99% amino acid identity between the *HERC5* and HERC-like N-terminals, and 74–84% amino acid identity between the HERC-like and *HERC6* C-terminals (**Figure 2A**, **Supplementary Figure 4**). To test whether this may be reminiscent of recombination in rodents and bats, we used the GARD program. We identified a significant recombination breakpoint located upstream from the spacer region at 1,103 bp in bats and 1,118 bp in rodents (**Figures 2A, B**). The phylogenetic analyses of the resulting fragments (identified by GARD) confirmed the recombination in both bats and rodents. Specifically, we found that the HERC-like 5’-fragment clustered with the *HERC5* gene (clade supported by a significant bootstrap), while the 3’-fragment grouped with the *HERC6* gene (**Figures 2C–F**). Taken together, our findings reveal that a similar ancient mechanism has independently led to the fixation of a *HERC*-like gene, which in fact is a chimeric HERC5/6 gene containing the *HERC5* RLD domain and the *HERC6* spacer and HECT domains in bats and rodents.

Moreover, by analyzing the phylogenetic trees of *HERCs* in bats and rodents (**Figures 2C–F**), we found that the 5’ fragment of *HERC5/6* was genetically closer to *HERC5* from the same species. This was not the case for the 3’ end, where all 3’ fragments of the *HERC5/6* genes significantly grouped together. Combined with several GARD analyses, this supports that recent recombinations further occurred between the RLD domains of *HERC5* and *HERC5/6* genes (**Figure 2G**).

### *HERC6*, but Not *HERC5*, Has Been Under Strong Positive Selection During Mammalian Evolution

We next investigated whether *HERC5* and *HERC6* have been under selective pressure in mammals.

**Figure 3:**
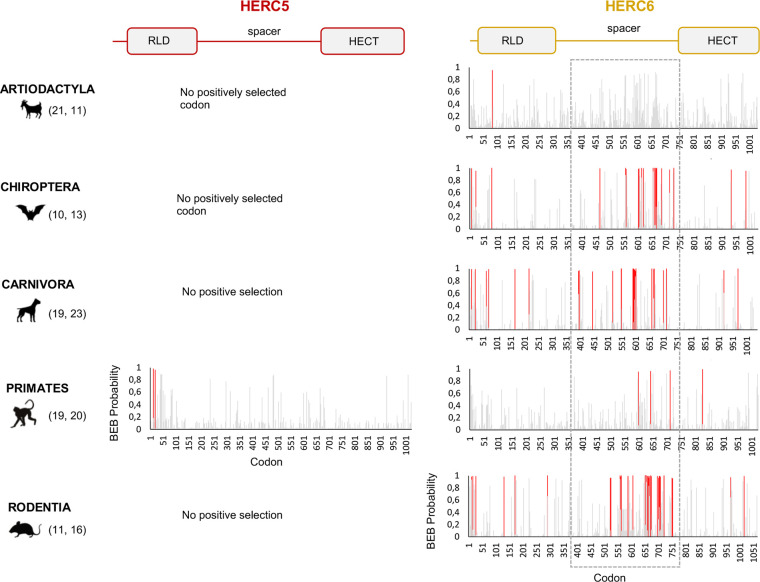
*HERC6*, and not *HERC5*, has experienced strong and mammalian-wide positive selection. Graphic panels represent the posterior probabilities of positive selection (Bayes empirical Bayes, BEB) (y axis) in the M2 Codeml model (allowing for positive selection, ω >1) for each codon (x axis) in *HERC5* (left) and *HERC6* (right) alignments. Red bars indicate the sites identified by both models, M2 and M8, with a BEB posterior probability greater than 0.95. Numbers in brackets are total species analyzed in each mammalian order for each gene. Site numbering is based on HERC5 protein sequences from *Homo sapiens*, HERC6 sequences from *Bos taurus*, *Rhinolophus ferrumequinum*, *Felis catus*, *Homo sapiens*, and *Mus musculus*. Above is a linear representation of HERC5 and HERC6 showing the structural domains, the RLD, spacer region, and HECT domains.

Our results revealed some signatures of positive selection in artiodactyl, primate, and bat *HERC5* (p-values <10^−3^), but none in rodent and carnivore species (p-value >0.5) (**Table 1**). At the codon level, the signal was overall very weak, with less than three positively selected codons assigned per order (posterior probability threshold fixed at 0.95, and p-value <0.05) (**Table 2**). In primates, two significant positively selected sites were identified in the RLD domain (**Figure 3**). Similarly, less than two codons evolved under positive selection in bats and artiodactyls (except for the MEME method which identified multiple positively selected sites in the artiodactyls), and none of the codons were common between methods (**Table 2**). Interestingly, the separate analysis of cetacean species revealed stronger signatures of positive selection at the gene (p-value = 6.10^−6^) and the codon levels (five sites identified by at least two methods), suggesting a lineage-specific adaptation.

**Table 1:** Positive selection analyses of mammalian *HERC5* and *HERC6* genes.

	Codeml M1 *vs* M2			Codeml M7 *vs* M8		
	p-value*^a^*	% of PSS*^b^*	M2 ω*^c^*	p-value*^a^*	% of PSS*^b^*	M8 ω*^c^*
HERC5						
Artiodactyls	0.0045	4.2	2.43	9.2E^−05^	10.3	1.9
Artiodactyls without cetaceans	0.0035	0.3	14.3	0.0024	0.3	13.6
Cetacea	4.4E^-06^	2.8	6.9	3.2E^−06^	2.9	6.9
Bats (1–192)	0.8423	–	–	0.3658	–	–
Bats (193–3,115)	0.0007	1	5.6	0.0002	1.3	4.8
Carnivores	0.6554	–	–	0.2083	–	–
Primates	0.0015	5.1	3.5	0.0009	5.6	3.4
Rodents (1–288)	1	–	–	1	–	–
Rodents (289–3,069)	1	–	–	0.2361	–	–
HERC6						
Artiodactyls whole gene	3.1E^−06^	6.1	3.7	2.1 E^−06^	7.4	3.4
Artiodactyls RLD domain	0.1071	–	–	0.0552	–	–
Artiodactyls Spacer region	3.9E^−05^	6.9	5	3.9E^−05^	7.8	4.6
Artiodactyls HECT domain	0.0029	1.1	3.6	0.0022	1. 3	3.2
Bats whole gene	1.4E^−40^	7	4.4	1.1 E^−44^	8	4.2
Bats RLD domain	3.4E^−06^	5.7	3.8	2.2E^−07^	7.2	3.4
Bats Spacer region	2.9E^−25^	10.1	4.5	2.4E^−27^	10.3	4.6
Bats HECT domain	1.6E^−10^	7.7	4.2	4.1E^−11^	9.4	3.9
Carnivores whole gene	3.2E^−43^	7	5.2	3E^−46^	7.5	5.1
Carnivores RLD domain	2.7E^−05^	8	3.3	1.5E^−05^	9.5	3.1
Carnivores Spacer region	1.5E^−33^	9.8	6.1	5.5E^−36^	10	6.1
Carnivores HECT domain	6E^−05^	4.7	4.7	2.4E^−05^	5.3	4.3
Primates whole gene	4.3E^−08^	4.9	4.2	1.8E^−08^	4.5	4.3
Primates RLD domain	0.9476	–	–	0.9016	–	–
Primates Spacer region	1.2E^−08^	8.4	5.4	1.3E^−08^	9.2	5.2
Primates HECT domain	1.1E^−06^	5.1	7.6	2E^−06^	5.2	7.6
Rodents whole gene	7.2E^−53^	9.1	3.7	1.3E^−60^	10.3	3.3
Rodents RLD domain	6.6E^−11^	5.1	3.9	4.2E^−13^	6.7	3.4
Rodents Spacer region	2.8E^−39^	14.1	4.2	2.7E^−41^	15	3.9
Rodents HECT domain	7.9E^−14^	6.8	4.5	7.6E^−16^	7.9	3.8
HERC5/6 paralog						
Rodents HECT domain	0.0003	20	2.1	0.0002	20	2.1

Results of positive selection analyses comparing models that disallow positive selection (M1 and M7) to models allowing positive selection (M2 and M8). ^a^p-values generated from maximum likelihood ratio tests indicate whether the model that allows positive selection (models M2 and M8) better fits the data than the nearly neutral one (M1 and M7). ^b^Percentage of codons evolving under positive selection (dN/dS ratio > 1 over the alignment). - not significant. cAverage dN/dS ratio associated with the classes K3 and K11, in the Codeml models M2 and M8, respectively, which allow positive selection.

**Table 2:** Positively selected codons in mammalian *HERC5*, *HERC6* and *HERC5/6* genes.

	MEME	FUBAR	Codeml M2	Codeml M8
**HERC5**				
Artiodactyls	6, 42, 63, 263, 408, 539, 565, 672, 696, 745, 992	39	–	189
Artiodactyls without Cetacea	–	39, 42, **552**, 555, 1034	–	**552**
Cetacea	**6**, 664, 666, **683**, **712**	**6**, 175, 347, **663**, **676**, **683**, **712**, 732, **782**	**663**, **676**	**6**, 662, **663**, **676**, **712**, **782**
Bats (193–2,913 bp)	22, 39,	550	–	654
Carnivores	89, 90, 91, 93, 250, 265, 298, 323, 568, 573	189, 401, 439, 840	–	–
Primates	–	**12**, **19**, **43**, 233, 296, **480**, **483**	**12**, **19**	**12**, **19**
Rodents (289–2,773 bp)	59, 76, 87, 98, **191**, 363, 462, 658, 870	**191**	–	–
**HERC6**				
Artiodactyls	56, 62, 112, **116**, 164, 167, 568, 661, 708, 723, 797	15, **116**, 599, 603, 655, 886		
Bats	**6**, 57, 58, 163, 382, 396, **549**, 552, 597, 600, **603**, **608**, 633, **646**, 650, **663**, 665, 671, **690**, **704**, **706**, 716, 814, 910, **911**, 995, 1014, 1015	**6**, **549**, **603**, **608**, **642**, **646**, **647**, **648**, **663**, **690**, **704**, **706**, **911**	**6**, **18**, **74**, **454**, **546**, **549**, **591, 595**, **603**, **608**, **642**, **646**, **647**, **648, 650, 651**, **663**, **690**, **706**, **911**, **964**	**6, 18, 74, 454**, **471,546, 549, 591, 595, 603, 608, 642, 643**, **646, 647, 648, 650, 651, 663, 690, 706, 911**, **964**, 974
Carnivores	30, 112, **166**, **216**, 225, **398**, **447**, 515, 517, **550**, 574, **593**, **596**, **600**, **602**, **603**, **659**, **666**, **669**, 753, 794, 846, 1023	**166**, **216**, 397, **398**, **447**, 549, **550**, **593**, 595, **600**, **602**, **603**, **659**, **666**, **669**, 713, 968	**9**, **23**, **63**, **70**, **166, 216**, 395, **396, 517, 547, 548, 591, 593, 594, 596, 598, 600, 601, 657, 699, 711, 917, 966**	**9, 23, 63, 70, 166, 216, 396, 517, 547, 548, 591, 593, 594, 596, 598, 600, 601, 657**, 664, 667**, 699, 711, 917, 966**
Primates	401, 472, 556, 647, 759, **828**, 1025	76, **597**, 631, 651, 700, **828**, 950, 989, 1021	**597**, **641**, **710**, **826**	370, 596, **597**, **641**, 649, 698, **710**, **826**, 987, 1020
Rodents	**10**, **11**, **16**, 59, 66, 123, 209, 268, 275, **317**, 328, 366, 450, 474, **546**, **548**, **550**, 554, **602, 606**, 609, **611**, **638, 642**, **643**, 644, 649, 677, **686**, **688**, 697, 700, 743, 756, 797, 838, 842, 872	**10**, **11**, **16, 287**, **546, 548, 550**, **551**, **557, 593, 594, 597**, **611, 642, 643**, **688**	**10, 16, 22, 125, 166, 287, 317, 513, 517, 547, 550, 551, 552, 553, 557, 593, 594, 597, 601, 602, 603, 605, 606, 610, 611, 613, 614, 635, 638, 642, 643, 645, 656, 657, 686, 688, 901, 952**	**10, 16, 22, 125, 166**, 219**, 287, 317, 513, 517, 547, 550, 551, 552, 553, 557, 593, 594, 597, 601, 602, 602, 605, 606, 610, 611, 613, 614, 635, 638, 642, 643, 645, 656, 657, 686, 688, 901, 952**
**HERC5-6 paralogs**				
Rodents	69, 138, 261, **409**, **467**, 774, 926	29, 120, 361, **467**, 506, 507, **518**, 556, **566**, **660**	**467**	22, **409**, **467**, **518**, **566**, **660**

Results from site-specific positive selection analyses, with a posterior probability (PP) of BEB >0.95 for the models M2 and M8 from Codeml, >0.9 for FUBAR, and a p-value <0.05 for MEME. Codons in bold are those that were assigned by at least two different methods. Codon numbering is based on HERC5 sequences from Bos taurus, Tursiops truncates, Phyllostomus discolor, Felis catus, Homo sapiens, and Dipodomys ordii, HERC6 sequences from Bos taurus, Rhinolophus ferrumequinum, Felis catus, Homo sapiens, and Mus musculus, HERC5/6 sequence from Fukomys damarensis.

**Figure 4:**
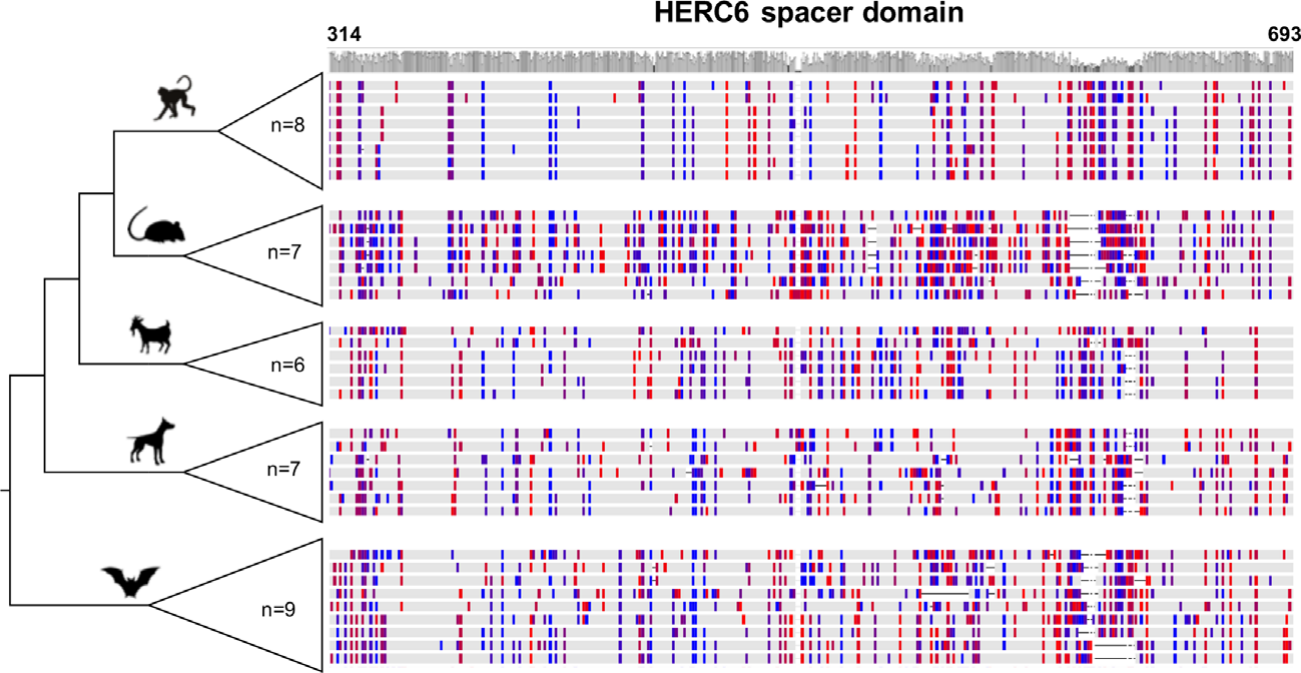
Rapid evolution of mammalian HERC6 spacer region is characterized by multiple amino acid changes and major indels. Multiple alignment and comparison of the HERC6 spacer region between and within mammalian orders. Left, cladogram with the number of sequences used for each clade (n = 6 to 9). Right, colors indicate site variations between the sequences as compared to the consensus sequence with a threshold of 25% (Geneious, Biomatters; blue/red, hydrophilic/hydrophobic residues), while gray represents similarity with the consensus. The average pairwise percentage of identity is graphically represented above in gray. The codon numbers are based on human HERC6 sequence.

In contrast, we found very strong signatures of ancient and recurrent positive selection in *HERC6*, at both the gene and the codon levels. All five mammalian orders exhibited a significant excess of non-synonymous rate along the *HERC6* coding sequences, specifically in bats, carnivores, and rodents (p-value <10^−43^ in bats, carnivores, and rodents; and p-value <10^−6^ in artiodactyls and primates). Positive selection was observed in each of the three domains of HERC6—the RLD domain, the spacer region, and the HECT domain—in bat, carnivore, and rodent species, while many of the signatures were concentrated in the spacer region and the HECT domains in primates and artiodactyls (**Table 1**, **Figure 3**). Remarkably, the fastest-evolving codons mapped into the *HERC6* spacer region of all five mammalian orders (p-value <10^−26^ in bats, carnivores, and rodents, p-value <10^−5^ in artiodactyls and primates). More than 14 sites were identified by at least two methods in bats, carnivores, and rodents, thereby constituting a hotspot of highly variable sites in mammalian *HERC6* (**Table 2** and **Figure 3**). In line with this finding, alignment of the spacer region revealed that it is an extremely divergent domain, characterized by multiple amino acid changes and indels between and within groups, in particular in rodents and bats (**Figure 4**).

Therefore, although *HERC5* presents low evidence of positive selection in mammals, *HERC6* has experienced very strong adaptive evolution. Both genes showed differential evolutionary profiles across/between mammals, with lineage-specific and domain-specific adaptations.

### The Rodent Chimeric *HERC5/6* Paralog Has Evolved Under Strong Positive Selection

We then addressed whether the newly identified chimeric *HERC5/6* gene, which contains the *HERC5* RLD domain and the *HERC6* spacer and HECT domains, has also experienced positive selection. As coding sequences from five rodent species were available, we assessed the inter-species evolutionary history of the chimeric *HERC5/6* gene within this group. Of note, there were insufficient bat sequences/species to perform the corresponding analyses. The likelihood ratio tests revealed significant positive selection in rodent *HERC5/6* (p-value <0.0003, **Table 2**). Importantly, all the positively selected codons mapped in the spacer region, and were concentrated between the amino acids 409 and 660 (**Figure 3**), suggesting that the spacer domain has been the target of strong positive selection as observed in HERC6.

### Rodent and Bat *HERC6* Genes Have Been Under Stronger Positive Selection Compared to Other Mammals

**Figure 5:**
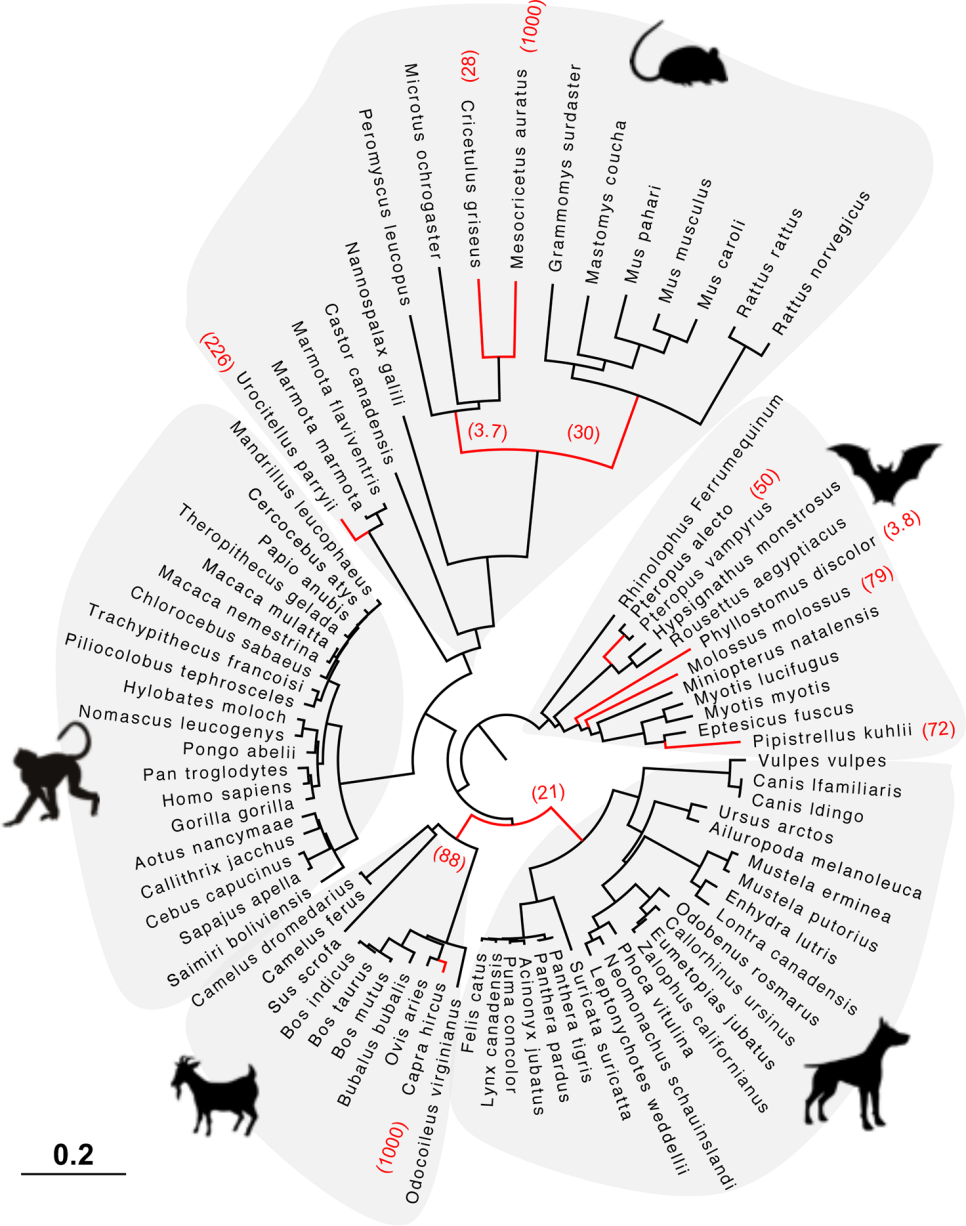
*HERC6* has been under strong positive selection during rodent and bat evolution. Maximum likelihood phylogenetic tree of mammalian HERC6 gene showing the branches under significant positive selection (p-value <0.05, in red) assigned by aBSREL from the HYPHY package. The numbers in brackets indicate the estimated values of the ω at the branch. The scale bar indicates the proportion of genetic variation.

Finally, we tested whether positive selection has differentially impacted the evolution of *HERC5* and *HERC6* across mammals. We found that the chiropteran and rodent *HERC6* genes have experienced intensive episodic positive selection compared to the other groups (**Figure 5**). In particular, five chiropteran lineages distributed along bat phylogeny (*Rhinolophus ferrumequinum*, *Pteropus* ancestral branch*, Phylllostomus discolor*, *Molossus molossus*, and *Pipistrellus kuhlii)* and five rodent branches (two ancestral branches of mouse related clade, *Cricetulus griseus*, *Mesocricetus auratus*, and *Urocitellus parryii*) have undergone a significant excess of amino acid changes, with a ratio ω >3.8. Differential selective pressure in *HERC5* was also evidenced across mammalian lineages, but to a lesser extent: only two branches were found under significant positive selection, in bats (*Hipposideros armiger*, ω = 189) and carnivores (*Ailuoropoda melanoleuca*, ω = 78).

## Discussion

Deciphering the evolutionary and functional diversification of the antiviral innate immunity in mammals is of primary importance to better understand modern viral pathogens, virus-host interfaces, and identify novel antiviral strategies. The functional significance of HERC5 and HERC6 is underlined by their ancient origin and conserved expression in vertebrates (25). However, their evolutionary history in mammals has remained unclear. Here, we have carried out in-depth phylogenetic and genomic analyses to address how mammalian *HERC5* and *HERC6* have evolved over millions of years of divergence.

### Differential Evolutionary Fate of *HERC5* Across Mammals

Although mammalian *HERC5* was previously reported as a rapidly evolving gene (33), we only found strong evidences of recurrent positive selection in primates. In particular, two codons in primate *HERC5* have rapidly evolved in the RLD domain, possibly reminiscent of pathogen-exerted pressure (3, 53–55). In line with this, blade 1 of the primate RLD domain was recently reported to be an important functional region for HERC5 anti-HIV antiviral activity (25). Thus, retroviruses may have played a role in the diversification of *HERC5* during primate evolution. Such patterns have been reported in many primate restriction factors, including *BST2* (56–58), *TRIM5* (10, 59–61), and *APOBEC3* (14, 62–64). Because the role of HERC5 is not limited to host defense against retroviruses, its evolution in primates may also reflect past selection against other viral pathogens such as influenza viruses and papillomaviruses.

In contrast, positive selection was solely evident at the gene level for artiodactyl and bat *HERC5*, and absent in rodents and carnivores. This pattern may be a result of lineage-specific selective drivers: differential viral exposure history, distinct mechanisms of viral antagonism, and/or may reflect overall pressure to maintain efficient cellular functions of HERC5. For example, apart from its antiviral role, some evidences suggest that HERC5 might be functionally involved in other pathways, such as spermatogenesis and cell cycle (22), as well as cancer (65). HERC5 may have thus evolved to maintain effective cellular functions rather than to escape viral antagonisms or to target viral pathogens in bats and artiodactyls. In mammals not exhibiting positive selection, viruses may have evolved indirect mechanisms of antagonism to counteract HERC5 function. In line with this, many viruses encode proteins that interfere with the ISGylation activity of HERC5, through direct interaction with the ISG15 protein (66–69). For example, the NS1B protein encoded by influenza virus antagonizes ISG15 conjugation through direct interaction (66).

### Accelerated Evolution of *HERC6* in Mammals

HERC6 is the only gene from the small *HERC* family exhibiting such high levels of adaptive changes with an extremely divergent spacer region in all mammals, except artiodactyls. Such rapid evolution of *HERC6*, with accumulated mutations replacing the amino acids and multiple amino acid insertions/deletions, most likely mirrors pathogen-driven adaptations as a result of past evolutionary arms-races (3, 53–55). This highlights a fundamental antiviral role for HERC6 in mammals. Previous functional evidences support that HERC6 is involved in mammalian antiviral immune response (28, 34, 35, 66). However, available studies have only focused on HERC6 anti-retroviral activity and ISGylation function. For example, HERC6 has been shown to be an IFN-inducible E3-ligase of ISG15 conjugation in mouse (28, 34, 35). In contrast, human HERC6 lacks the ISGylation activity, but it potently inhibits the primate lentivirus SIVmac viral production (25).

Specifically, we identified the spacer region as a potential pathogen—HERC6 interface, involved in the recognition of viral proteins and/or being the target of viral antagonists. Up to now, most studies have been devoted to the functional characterization of the RLD and HECT domains of HERC proteins, as they belong to the well-characterized protein families, RCC1 and E3-ligases, respectively. In contrast, the structural characteristic of the spacer region has not been related to any known protein, which hinders its description and functional role. However, its propensity for amino acid insertions/deletions and accumulated non-synonymous mutations, including changes with strong chemicophysical differences highlights a strong evolutionary plasticity. Such a hotspot of variability in unstructured regions has been reported for several restriction factors, such as MX1, in which the highly variable L4 loop has led to differential virus-host interfaces and plasticity (70, 71).

It is noteworthy that the RLD and HECT domains of *HERC6* were also subjected to positive selection in bats, carnivores, and rodents. These signatures may reflect different virus-host interfaces. This pattern was reported in the primate PKR protein, in which signatures of pathogen-driven selection are scattered along the protein as a result of interactions with multiple viral antagonists (72, 73). However, it is also possible that all positively selected sites cluster in a same spatial region of the protein. A 3D structural analysis of the *HERC6* protein would help assessing how the rapidly evolving sites are located in the protein, and allow determining whether *HERC6* presents multiple or unique host-pathogen interface(s). Up to now, the 3D structure has only been solved for the HECT domain and RLD domain separately [e.g (74, 75)]. Therefore, how the spacer region connects the HECT and RLD domains in a 3D structural dimension is currently unknown. Further studies on small HERC protein structure would help to better understand how the high variability in *HERC6* impacts its structure and function.

We found differential genetic profiles of *HERC6* across mammalian orders. The strongest signal was found in bats, carnivores, and rodents, suggesting that different strength/intensity of selective pressures have shaped the evolution of *HERC6* in mammals. This was confirmed by the branch-specific analyses, in which rodents and bats exhibit significant lineage-specific adaptive changes. Rodents and bats are the two most diverse mammalian orders, and host the highest viral richness among mammals (38). Both orders have thus been exposed to a greater viral diversity, compared to primates and artiodactyls. This may have increased the strength and extent of selective pressure exerted on the HERC6 protein.

### Unequal Recombination Has Led to the Duplication of a Chimeric *HERC5/6* in Rodents and Bats

Lineage-specific expansions of multigene families have shaped and complexified the mammalian antiviral repertoire over million years of evolution. Consequently, many unrecognized genes encoding for antiviral proteins are yet to be discovered. Here, we have identified duplications of a *HERC* paralog in the rodent Hystricognathi infra-order and the chiropteran *Myotis* genus, which has occurred around 30 MYA (76, 77) and 44 MYA (78–80), respectively.

Interestingly, these paralogs are chimeric *HERC5/6* genes coding for the HERC5 RLD fused to the *HERC6* spacer region and HECT domain. This suggests that an independent duplication has occurred through a similar mechanism in bats and rodents. Gene duplication can occur by different modes, mainly including unequal crossing over, retroposition, or chromosomal duplication (81, 82). In the former case, duplicated genes are physically linked in the chromosome, and can contain a fragment of a gene, a whole gene, or several genes (81). In contrast, retroposition engenders a retrotranscribed complementary DNA incorporated into the genome, which generally lacks intronic regions and regulatory sequences (81). Given that the *HERC5/6* are located in the canonical locus of *HERC5* and *HERC6* and contain the parental intronic regions, they have most likely resulted from an unequal crossing over between *HERC5* and *HERC6*.

This hypothesis is supported by the recombination analyses, which detected a significant breakpoint upstream of the spacer region in both mammalian orders. The fact that the recombination occurred at the same genetic location can be explained by two different, but not necessarily mutually exclusive, hypotheses. First, the recombination event can only occur at this location because of genomic structural constraints (i.e. genomic homology between paralogs). Second, the HERC6 spacer region and HECT domains are required for functional HERC5/6 proteins. The best example of such tandem duplication with domain fusion is the lineage-specific expansion of the *APOBEC3* family in mammals. Originating from an ancestral *APOBEC3* gene, tandem duplications as well as retrocopying events have radically expanded the repertoire of mammalian *APOBEC3* genes (14, 20). In primates, several of the *APOBEC3* genes have most likely resulted from the fusion of A3 domains, while the murine genome encodes a unique *APOBEC3Z2-APOBEC3Z3* fused gene (83), highlighting lineage specific functional adaptations.

Such gene duplications accompanied with gene fusion are major genetic innovations that functionally diversify the antiviral arsenal (3, 4, 7). For example, the expansion of the *APOBEC3* family has functionally diversified the antiviral activities and specificity of targeted viruses in primates (20, 62, 84). Given the antiviral role of HERC5 and the potential implication of HERC6 in antiviral immunity, we hypothesize that the HERC5/6 paralog provides a functional advantage against pathogenic viruses. This is supported by both the extremely rapid episodic evolution of *HERC6* in rodents and bats, and the signatures of positive selection in rodent *HERC5/6*. The fused *HERC5/6* gene may have evolved combined functional features of *HERC5* and *HERC6*. Alternatively, it may have retained the functional implication of the HERC5 RLD domain, but has functionally diverged from HERC6.

This latter hypothesis is more likely as we found that the RLD domain of HERC5 and of HERC5/6 are highly similar and cluster together within species (more than 95% pairwise amino acid identity). This pattern may reflect ongoing gene conversions between the RLD domains of *HERC5* and *HERC5/6*, thereby maintaining a N-terminal similar to the parental HERC5 protein.

Whether the independent fixation of the *HERC5/6* paralog in rodents and bats is a functional/phenotypic convergent evolutionary event has to be investigated. Moreover, it raises the question why both lineages have undergone such genetic innovation. While this duplication could be hazardous, it is possible that the rodent Hystricognathi infra-order and the bat *Myotis* genus share a common selective pressure, such as a viral pathogen family. Other forces such as ecological and/or environmental factors may have played a role, such as life-history traits or biodiversity changes.

### Perspectives

Studying the genetic adaptations of host innate immunity can provide insights into the evolutionary and functional determinants of host antiviral response. This approach has been a powerful tool for assessing the functional diversification of virus-host interfaces in many systems [e.g (59, 62, 85, 86)]. In this present study, we identified *HERC6* and *HERC5/6* as potential unrecognized restriction factors in mammals. Further functional investigations are now required to (i) decipher the antiviral function of *HERC6* and HERC5/6, (ii) determine how the accumulated variability in the spacer domain may impact their structure, function, and stability, (iii) unravel HERC6 binding interface with potential viral antagonists or targeted viral proteins, and (iv) determine on the other side which pathogens are targeted by those proteins. It will also be interesting to investigate whether and how the evolution of *HERCs* may impact the cellular partners involved in the ISGylation functions. For example, although we found that ISG15 is mostly conserved in mammals, it bears important variations at the C-terminal (data not shown). This could reflect host protein-protein co-evolutions or indirect viral antagonism of HERC restriction. Moreover, because the *HERCs* have been mostly studied in primates and rodents, it is possible that other proteins than ISG15 are involved in the ISGylation function in other mammals. In addition, deciphering whether and how the *HERC5/6* paralogs afford a selective advantage against pathogens in rodent and bat lineages is of main interest in virology and immunology fields, as both orders are important reservoirs of zoonotic pathogens. Finally, studying the functional implications of *HERC6* adaptation in mammals will not only allow to better understand how pathogens have shaped host immunity, but will provide important insights into the overlooked role of small HERC proteins in mammalian antiviral response.

Altogether, our results represent avenues for future functional studies of importance in mammalian innate immunity.

## Data Availability Statement

Publicly available datasets were analyzed in this study. This data can be found here: NCBI (https://www.ncbi.nlm.nih.gov/) and accession numbers are available in **Supplementary Table 1**.

## Author Contributions

SJ carried out the analyses of the data. SJ, LE, and DP performed the investigations. SJ, LE, and DP wrote the original draft of the paper. LE and DP acquired the funding, and coordinated the study. All authors contributed to the article and approved the submitted version.

## Funding

This work is funded by the ANR LABEX ECOFECT (ANR-11-LABX-0048 of the Université de Lyon, within the program Investissements d’Avenir [ANR-11-IDEX-0007] operated by the French National Research Agency). LE is further supported by the CNRS and by grants from amfAR (Mathilde Krim Phase II Fellowship no. 109140-58-RKHF), the Fondation pour la Recherche Médicale (FRM Projet Innovant no. ING20160435028), the FINOVI (“recently settled scientist” grant), the ANRS (no. ECTZ19143 and ECTZ118944), and a JORISS incubating grant. DP is supported by the CNRS, the European Regional Development Fund (ERDF) 2018-4512410/convention P-2020-BAFE-9 and 2018-4729510/convention P-2020-BAFE-23, and the ANR EBOFAC.

## Conflict of Interest

The authors declare that the research was conducted in the absence of any commercial or financial relationships that could be construed as a potential conflict of interest.

## Supplementary Material

Supplementary Figure 1 Maximum likelihood phylogenetic tree generated with the whole coding sequences of *HERC5*, *HERC6*, and *HERC3* nucleotide alignment from artiodactyl, carnivore, rodent, bat, and primate species. Asterisks indicate bootstrap values greater than 80%. The scale bar represents the proportion of genetic variation (0.2 for the scale), and is indicated at the bottom. Sequences are collapsed in each order for better readability.

Supplementary Figure 2 Pseudogenization of cetacean *HERC6* **(A)**. Multiple amino acid alignment of six cetacean *HERC6* sequences showing multiple substitutions, insertions, and deletions **(B).** Multiple nucleotide alignment with corresponding amino acids of cetacean, rodent, bat, ruminant, primate, and carnivore *HERC6*, highlighting a conserved stop codon in the cetacean species (codon 174 in *Balaenoptera acutorostrata*). Nucleotide and amino acid sequences are shown using Geneious.

Supplementary Figure 3 Maximum likelihood phylogenetic tree generated with the whole coding sequences of *HERC5*, *HERC6*, *HERC5/6*, and *HERC3* nucleotide alignment in bats (left) and rodents (right). The chimeric duplicated *HERC5/6* genes are shown in red. Asterisks indicate bootstrap values greater than 80%. The scale bar at 0.2 is indicated below.

Supplementary Figure 4 Alignment of the protein sequence of HERC5, HERC5/6, and *HERC6* from bats and rodents. The percentages of pairwise amino acid identities between the N-terminals of HERC5/6 and HERC5 or HERC6, as well as the C-terminals of HERC5/6 and HERC5 or HERC6 are indicated. The significant recombination breakpoints (red arrows, p-value <0.05) assigned by GARD program are shown for bat and rodent *HERC5/6* gene. Because the coding sequence of HERC6 gene from *Heterocephalus glaber* was incomplete with many missing data, it was not included in the figure. Likewise, some portions of the N-terminal of HERC5, as well as the C-terminals, from HERC5/6 and HERC6 are missing in the protein alignment of the chiropteran species, *Myotis brandtii* (top).

Supplementary Table 1 Information on publicly available datasets analyzed in this study. Accession numbers are available in NCBI (https://www.ncbi.nlm.nih.gov/).
