# Supplementary figures and images for: Rapid Evolution of HERC6 and Duplication of a Chimeric HERC5/6 Gene in Rodents and Bats Suggest an Overlooked Role of HERCs in Mammalian Immunity
Source: Front Immunol. 2020 Dec 18;11:605270. doi: 10.3389/fimmu.2020.605270 (PMC7775381; doi:10.3389/fimmu.2020.605270)

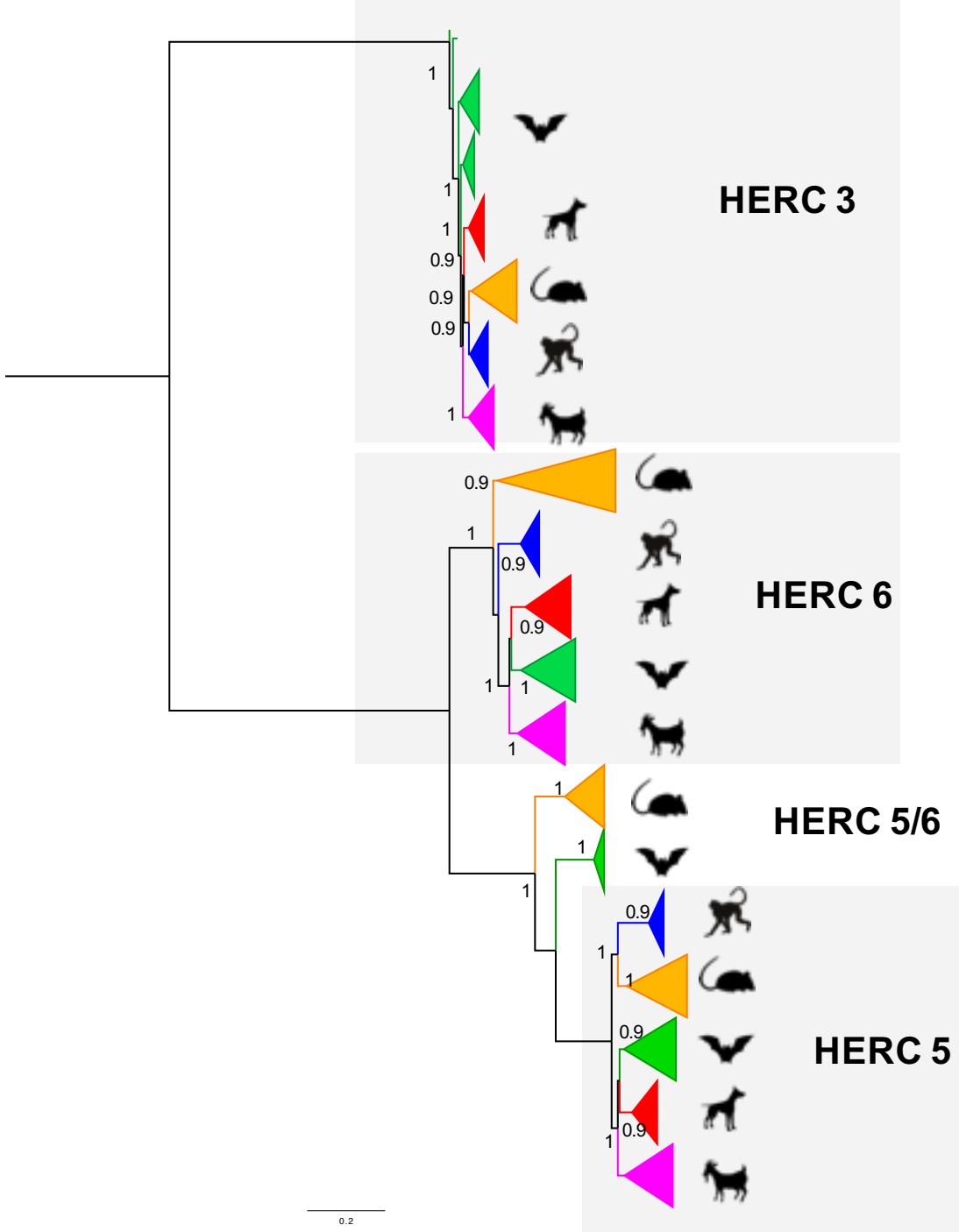

Supplement: Supplementary Figure 1 — Maximum likelihood phylogenetic tree generated with the whole coding sequences of HERC5, HERC6, and HERC3 nucleotide alignment from artiodactyl, carnivore, rodent, bat, and primate species. Asterisks indicate bootstrap values greater than 80%. The scale bar represents the proportion of genetic variation (0.2 for the scale), and is indicated at the bottom. Sequences are collapsed in each order for better readability. [file Image_1.pdf]
