# Supplementary material for: Rapid Evolution of HERC6 and Duplication of a Chimeric HERC5/6 Gene in Rodents and Bats Suggest an Overlooked Role of HERCs in Mammalian Immunity
Source: Front Immunol. 2020 Dec 18;11:605270. doi: 10.3389/fimmu.2020.605270 (PMC7775381; doi:10.3389/fimmu.2020.605270)
Supplement: Supplementary Figure 2 — Pseudogenization of cetacean HERC6 (A). Multiple amino acid alignment of six cetacean HERC6 sequences showing multiple substitutions, insertions, and deletions (B). Multiple nucleotide alignment with corresponding amino acids of cetacean, rodent, bat, ruminant, primate, and carnivore HERC6, highlighting a conserved stop codon in the cetacean species (codon 174 in Balaenoptera acutorostrata). Nucleotide and amino acid sequences are shown using Geneious. [file Image_2.pdf]

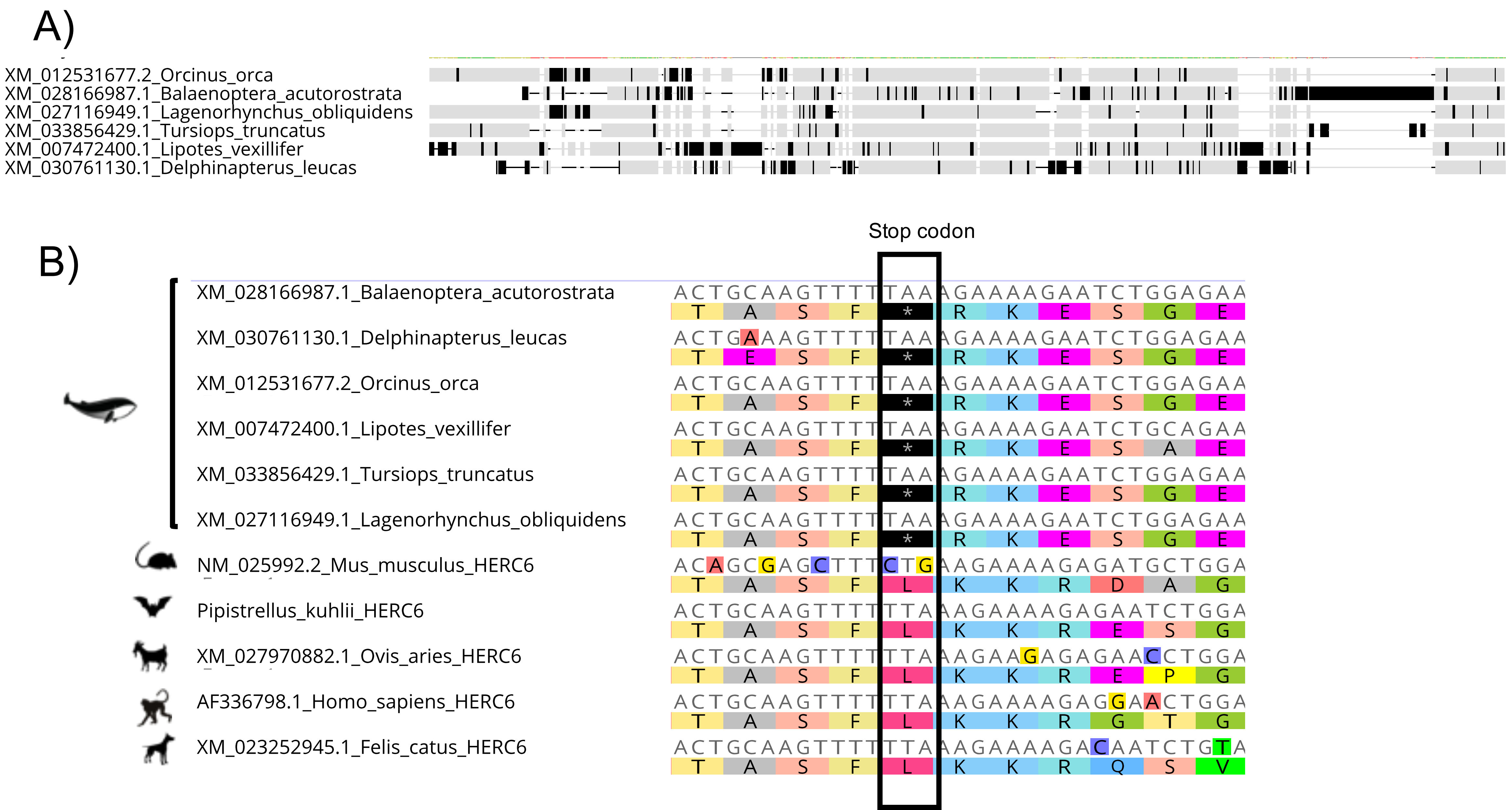
A)
XM_012531677.2_Orcinus_orca
XM_028166987.1_Balaenoptera_acutorostrata
XM_027116949.1_Lagenorhynchus_obliquidens
XM_033856429.1_Tursiops_truncatus
XM_007472400.1_Lipotes_vexillifer
XM_030761130.1_Delphinapterus_leucas
Stop codon
B)
XM_028166987.1_Balaenoptera_acutorostrata ACTGCAAGTTTTTAAAGAAAAGAATCTGGAGAA
T A S F * R K E S G E
XM_030761130.1_Delphinapterus_leucas ACTGAAAGTTTTTAAAGAAAAGAATCTGGAGAA
T E S F * R K E S G E
XM_012531677.2_Orcinus_orca ACTGCAAGTTTTTAAAGAAAAGAATCTGGAGAA
T A S F * R K E S G E
XM_007472400.1_Lipotes_vexillifer ACTGCAAGTTTTTAAAGAAAAGAATCTGCAGAA
T A S F * R K E S A E
XM_033856429.1_Tursiops_truncatus ACTGCAAGTTTTTAAAGAAAAGAATCTGGAGAA
T A S F * R K E S G E
XM_027116949.1_Lagenorhynchus_obliquidens ACTGCAAGTTTTTAAAGAAAAGAATCTGGAGAA
T A S F * R K E S G E
NM_025992.2_Mus_musculus_HERC6 ACAGCGAGCTTTCTGAAGAAAAGAGATGCTGGA
T A S F L K K R D A G
Pipistrellus_kuhlii_HERC6 ACTGCAAGTTTTTTAAAGAAAAGAGAATCTGGA
T A S F L K K R E S G
XM_027970882.1_Ovis_aries_HERC6 ACTGCAAGTTTTTTAAAGAAGAGAGAACCTGGA
T A S F L K K R E P G
AF336798.1_Homo_sapiens_HERC6 ACTGCAAGTTTTTTAAAGAAAAGAGGAACTGGA
T A S F L K K R G T G
XM_023252945.1_Felis_catus_HERC6 ACTGCAAGTTTTTTAAAGAAAAGACAATCTGTA
T A S F L K K R Q S V
