# Supplementary material for: Rapid Evolution of HERC6 and Duplication of a Chimeric HERC5/6 Gene in Rodents and Bats Suggest an Overlooked Role of HERCs in Mammalian Immunity
Source: Front Immunol. 2020 Dec 18;11:605270. doi: 10.3389/fimmu.2020.605270 (PMC7775381; doi:10.3389/fimmu.2020.605270)
Supplement: Supplementary Figure 3 — Maximum likelihood phylogenetic tree generated with the whole coding sequences of HERC5, HERC6, HERC5/6, and HERC3 nucleotide alignment in bats (left) and rodents (right). The chimeric duplicated HERC5/6 genes are shown in red. Asterisks indicate bootstrap values greater than 80%. The scale bar at 0.2 is indicated below. [file Image_3.pdf]

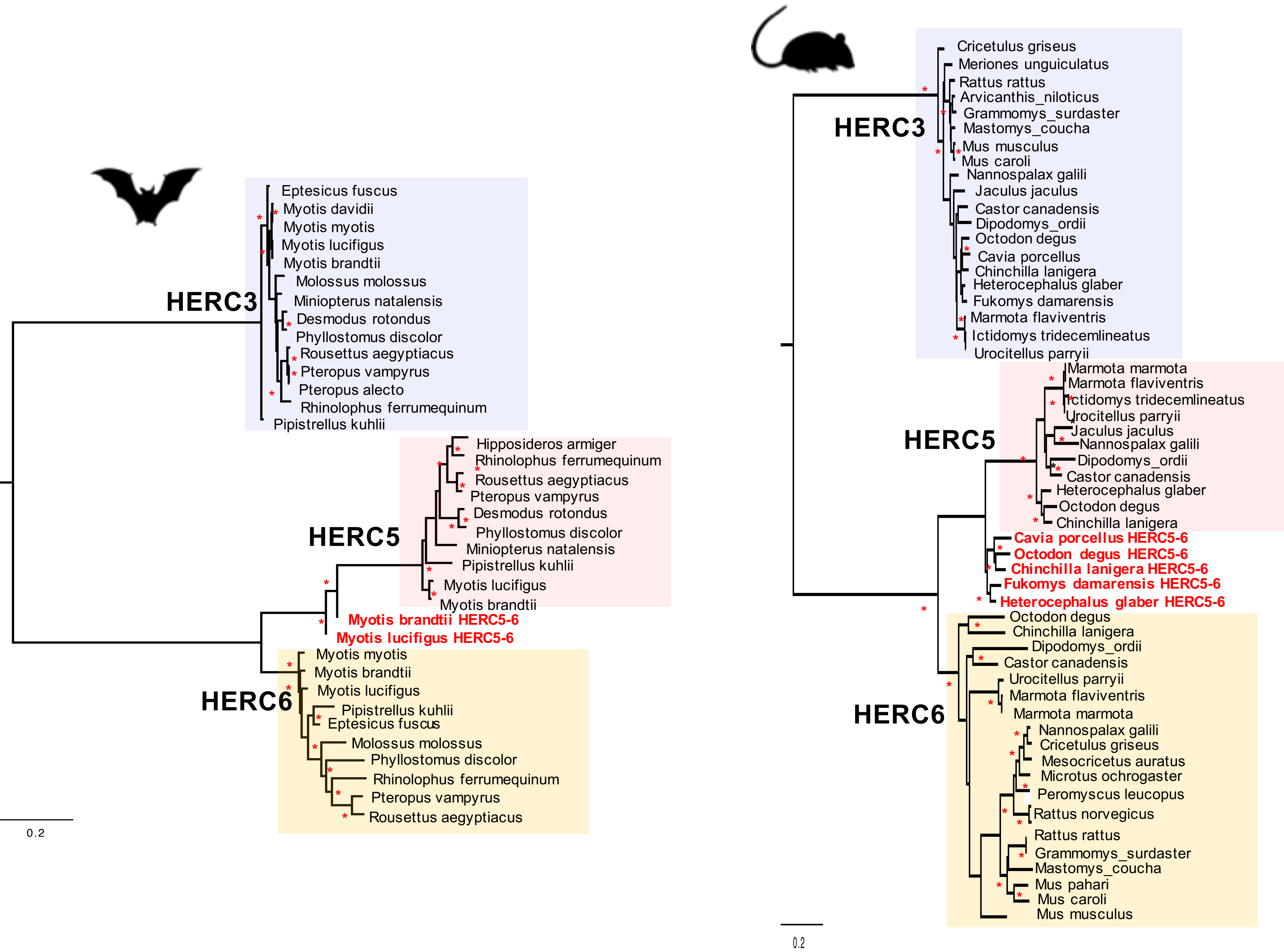
HERC3
Eptesicus fuscus
Myotis davidii
Myotis myotis
Myotis lucifigus
Myotis brandtii
Molossus molossus
Miniopterus natalensis
Desmodus rotondus
Phyllostomus discolor
Rousettus aegyptiacus
Pteropus vampyrus
Pteropus alecto
Rhinolophus ferrumequinum
Pipistrellus kuhlii
HERC5
Hipposideros armiger
Rhinolophus ferrumequinum
Rousettus aegyptiacus
Pteropus vampyrus
Desmodus rotondus
Phyllostomus discolor
Miniopterus natalensis
Pipistrellus kuhlii
Myotis lucifigus
Myotis brandtii
Myotis brandtii HERC5-6
Myotis lucifigus HERC5-6
HERC6
Myotis myotis
Myotis brandtii
Myotis lucifigus
Pipistrellus kuhlii
Eptesicus fuscus
Molossus molossus
Phyllostomus discolor
Rhinolophus ferrumequinum
Pteropus vampyrus
Rousettus aegyptiacus
0.2
HERC3
Cricetulus griseus
Meriones unguiculatus
Rattus rattus
Arvicanthis_niloticus
Grammomys_surdaster
Mastomys_coucha
Mus musculus
Mus caroli
Nannospalax galili
Jaculus jaculus
Castor canadensis
Dipodomys_ordii
Octodon degus
Cavia porcellus
Chinchilla lanigera
Heterocephalus glaber
Fukomys damarensis
Marmota flaviventris
Ictidomys tridecemlineatus
Urocitellus parryii
HERC5
Marmota marmota
Marmota flaviventris
Ictidomys tridecemlineatus
Urocitellus parryii
Jaculus jaculus
Nannospalax galili
Dipodomys_ordii
Castor canadensis
Heterocephalus glaber
Octodon degus
Chinchilla lanigera
Cavia porcellus HERC5-6
Octodon degus HERC5-6
Chinchilla lanigera HERC5-6
Fukomys damarensis HERC5-6
Heterocephalus glaber HERC5-6
HERC6
Octodon degus
Chinchilla lanigera
Dipodomys_ordii
Castor canadensis
Urocitellus parryii
Marmota flaviventris
Marmota marmota
Nannospalax galili
Cricetulus griseus
Mesocricetus auratus
Microtus ochrogaster
Peromyscus leucopus
Rattus norvegicus
Rattus rattus
Grammomys_surdaster
Mastomys_coucha
Mus pahari
Mus caroli
Mus musculus
0.2
