# Supplementary material for: Rapid Evolution of HERC6 and Duplication of a Chimeric HERC5/6 Gene in Rodents and Bats Suggest an Overlooked Role of HERCs in Mammalian Immunity
Source: Front Immunol. 2020 Dec 18;11:605270. doi: 10.3389/fimmu.2020.605270 (PMC7775381; doi:10.3389/fimmu.2020.605270)
Supplement: Supplementary Figure 4 — Alignment of the protein sequence of HERC5, HERC5/6, and HERC6 from bats and rodents. The percentages of pairwise amino acid identities between the N-terminals of HERC5/6 and HERC5 or HERC6, as well as the C-terminals of HERC5/6 and HERC5 or HERC6 are indicated. The significant recombination breakpoints (red arrows, p-value <0.05) assigned by GARD program are shown for bat and rodent HERC5/6 gene. Because the coding sequence of HERC6 gene from Heterocephalus glaber was incomplete with many missing data, it was not included in the figure. Likewise, some portions of the N-terminal of HERC5, as well as the C-terminals, from HERC5/6 and HERC6 are missing in the protein alignment of the chiropteran species, Myotis brandtii (top). [file Image_4.pdf]

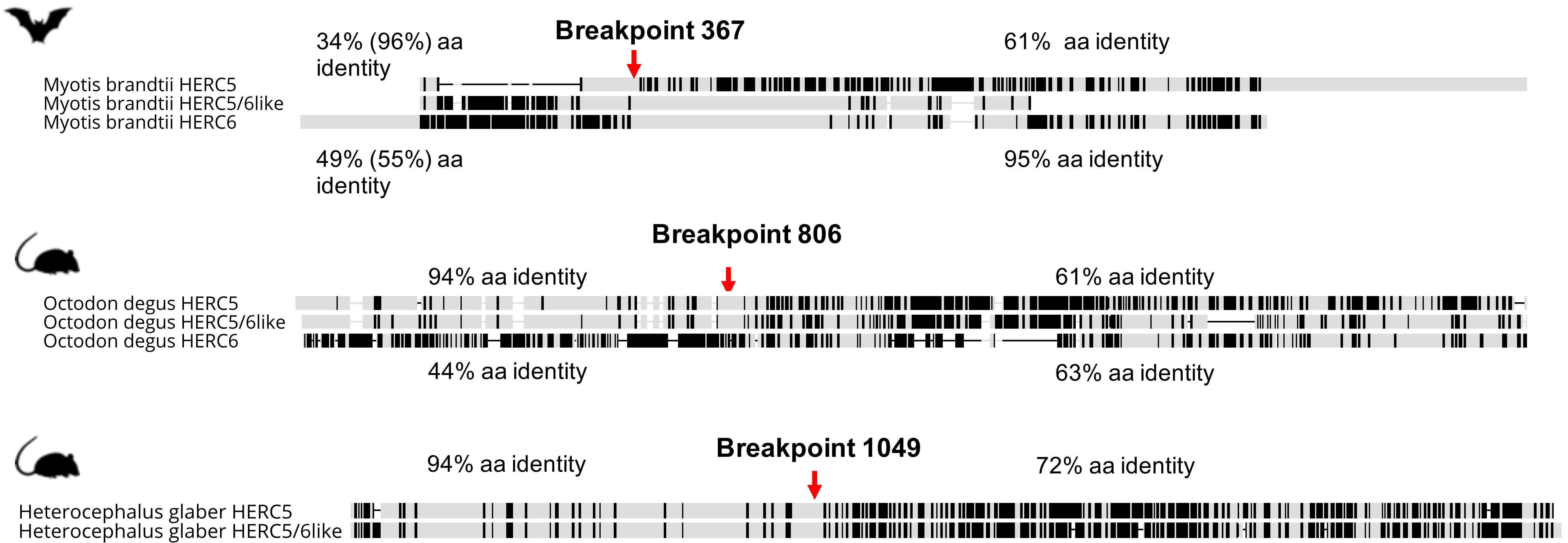
Breakpoint 367
34% (96%) aa identity
61% aa identity
Myotis brandtii HERC5
Myotis brandtii HERC5/6like
Myotis brandtii HERC6
49% (55%) aa identity
95% aa identity
Breakpoint 806
94% aa identity
61% aa identity
Octodon degus HERC5
Octodon degus HERC5/6like
Octodon degus HERC6
44% aa identity
63% aa identity
Breakpoint 1049
94% aa identity
72% aa identity
Heterocephalus glaber HERC5
Heterocephalus glaber HERC5/6like
