# Supplementary material for: Rapid Evolution of HERC6 and Duplication of a Chimeric HERC5/6 Gene in Rodents and Bats Suggest an Overlooked Role of HERCs in Mammalian Immunity
Source: Front Immunol. 2020 Dec 18;11:605270. doi: 10.3389/fimmu.2020.605270 (PMC7775381; doi:10.3389/fimmu.2020.605270)
Supplement: Supplementary Table 1 — Information on publicly available datasets analyzed in this study. Accession numbers are available in NCBI (https://www.ncbi.nlm.nih.gov/). [file Table_1.docx]

| **Species names** | **HERC5 accession numbers** | **HERC6 accession numbers** | **Scaffolds** |
| --- | --- | --- | --- |
| **Artiodactyls** |  |  |  |
| *Bos_indicus* |  | XM_019962473.1 | Bos indicus chromosome 6, Bos_indicus_1.0 |
| *Bos_mutus* |  | XM_005897788.2 | Bos mutus yakQH1, BosGru_v2.0 scaffold957_1 |
| *Bos_taurus* | XM_005207769.4 | XM_010806029.3 | Bos taurus L1 Dominette 01449 chromosome 6, ARS-UCD1.2 |
| *Bison_bison* | XM_010860169.1 |  | Bison bison, Bison_UMD1.0 scf7180017864464 |
| *Bubalus_bubalis* | XM_025290426.1 | XM_006043251.2 | Bubalus bubalis chromosome 7, ASM312139v1 |
| *Camelus_bactrianus* | XM_010966131.1 | XM_010966133.1 | Camelus bactrianus , Ca_bactrianus_MBC_1.0 scaffold384 |
| *Camelus_dromedarius* | XM_031433767.1 | XM_031433801.1 | Camelus dromedarius Drom800 breed African chromosome 2, CamDro3 |
| *Camelus_ferus* | XM_032498192.1 | XM_032498214.1 | Camelus ferus YT-003-E chromosome 2, BCGSAC_Cfer_1.0 |
| *Capra_hircus* | XM_018049314.1 | XM_018049316.1 | Capra hircus breed San Clemente chromosome 6, ASM170441v1 |
| *Odocoileus_virginianus* | XM_020868995.1 | XM_020868996.1 | Odocoileus virginianus texanus, Ovir.te_1.0 scaffold424 |
| *Ovis_aries* | XM_012179691.3 | XM_027970882.1 | Ovis aries strain OAR_USU_Benz2616 chromosome 6 |
| *Sus_scrofa* | XM_021100766.1 | XM_021100762.1 | Sus scrofa TJ Tabasco breed Duroc chromosome 8, Sscrofa11.1 |
| *Balaenoptera_acutorostrata* |  |  | Balaenoptera acutorostrata BalAcu1.0 scaffold225 |
| *Delphinapterus_leucas* | XM_022582308.2 |  | Delphinapterus leucas ASM228892v3 scaffoldscaffold14 |
| *Globicephala_melas* | XM_030865688.1 |  | Globicephala melas ASM654740v1 scaffold28 |
| *Lagenorhynchus_obliquidens* | XM_027116896.1 |  | Lagenorhynchus obliquidens ASM367639v1 scaffold17 |
| *Lipotes_vexillifer* | XM_007450670.1 |  | Lipotes_vexillifer_v1 scaffold34 |
| *Monodon_monoceros* | XM_029235466.1 |  | Monodon monoceros NGI_Narwhal_1 Super_Scaffold_20 |
| *Neophocaena_asiaeorientalis* | XM_024736148.1 |  | Neophocaena_asiaeorientalis_V1 scaffold7 |
| *Orcinus_orca* | XM_004266128.3 |  | Orcinus orca Morgan Oorc_1.1 Scaffold17 |
| *Phocoena_sinus* | XM_032632262.1 |  | Phocoena sinus mPhoSin1 chromosome 5, mPhoSin1.pri |
| *Physeter_catodon* | XM_024126675.2 |  | Physeter catodon SW-GA chromosome 7, ASM283717v2 |
| *Tursiops_truncatus* | XM_019926409.2 |  | Tursiops truncatus mTurTru1 chromosome 5, mTurTru1.mat.Y |
| *Vicugna_pacos* | XM_015250520.2 |  | Vicugna pacos Carlotta (AHFN-0088) chromosome 2 , VicPac3.1 |
| **Bats** |  |  |  |
| *Desmodus_rotundus* | XM_024574779.1 | XM_024574779.1 | Desmodus rotundus DRU21DN04 , ASM294091v2 ScWXtkA_64 |
| *Eptesicus_fuscus* |  | XM_008143693.2 | Eptesicus fuscus BU_THK_EF1 , EptFus1.0 scaffold00022 |
| *Hipposideros_armiger* | XM_019633253.1 |  | Hipposideros armiger ML-2016 , ASM189008v1 |
| *Hypsignathus_monstrosus* | GHDN01002874.1 | GHDN01002875.1 |  |
| *Miniopterus_natalensis* | XM_016203215.1 | XM_016203231.1 | Miniopterus natalensis MN2012-01 , Mnat.v1 scaff239 |
| *Molossus molossus* |  | [HLmolMol2](https://genome-public.pks.mpg.de/cgi-bin/hgTracks?db=HLmolMol2) |  |
| *Myotis myotis* |  | [HLmyoMyo6](https://genome-public.pks.mpg.de/cgi-bin/hgTracks?db=HLmyoMyo6) |  |
| *Myotis_lucifugus* | XM_006084661.3 | XM_014452069.2 | Myotis lucifugus , Myoluc2.0 scaffold_12 |
| *Myotis_brandtii* |  |  | Myotis brandtii , ASM41265v1 scaffold1403 |
| *Phyllostomus discolor* | [HLphyDis3](https://genome-public.pks.mpg.de/cgi-bin/hgTracks?db=HLphyDis3) | [HLphyDis3](https://genome-public.pks.mpg.de/cgi-bin/hgTracks?db=HLphyDis3) | Phyllostomus discolor chromosome 3, mPhyDis1_v1.p |
| *Pipistrellus kuhlii* | [HLpipKuh2](https://genome-public.pks.mpg.de/cgi-bin/hgTracks?db=HLpipKuh2) | [HLpipKuh2](https://genome-public.pks.mpg.de/cgi-bin/hgTracks?db=HLpipKuh2) |  |
| *Pteropus_alecto* |  | XM_006910719.3 | Pteropus alecto , ASM32557v1 scaffold191 |
| *Pteropus_vampyrus* | XM_011356307.2 | XM_011356307.2 | Pteropus vampyrus Shadow , Pvam_2.0 Scaffold818 |
| *Rhinolophus ferrumequinum* | [HLrhiFer5](https://genome-public.pks.mpg.de/cgi-bin/hgTracks?db=HLrhiFer5) | [HLrhiFer5](https://genome-public.pks.mpg.de/cgi-bin/hgTracks?db=HLrhiFer5) | Rhinolophus ferrumequinum chromosome 19, mRhiFer1_v1.p |
| *Rousettus_aegyptiacus* | [HLrouAeg4](https://genome-public.pks.mpg.de/cgi-bin/hgTracks?db=HLrouAeg4) | XM_016161038.1 | Rousettus aegyptiacus 1219 , Raegyp2.0 |
| **Carnivores** |  |  |  |
| *Acinonyx_jubatus* | XM_027060637.1 | XM_027060657.1 | Acinonyx jubatus Rico , Aci_jub_2_ph1_scaffold0 |
| *Ailuropoda_melanoleuca* | XM_011232575.2 | XM_019806094.1 | Ailuropoda melanoleuca Jingjing chromosome 11, ASM200744v2 |
| *Callorhinus_ursinus* | XM_025873413.1 | XM_025873415.1 | Callorhinus ursinus ASM326570v1 scaffold107 |
| *Canis_lupus dingo* | XM_025425126.1 | XM_025425172.1 | Canis lupus dingo Sandy , ASM325472v1 001672F_SuperScaffold_82 |
| *Canis_lupus_familiaris* |  | XM_005639167.3 | Canis lupus familiaris breed boxer chromosome 32, CanFam3.1 |
| *Enhydra_lutris* |  | XM_022512204.1 | Enhydra lutris kenyoni, ASM228890v2 scaffoldscaffold3 |
| *Eumetopias_jubatus* | XM_028117745.1 | XM_028117746.1 | Eumetopias jubatus , ASM402803v1 scaffold85 |
| *Felis_catus* | XM_023252942.1 | XM_023252945.1 | Felis catus chromosome B1, Felis_catus_9.0 |
| *Leptonychotes_weddellii* | XM_031023593.1 | XM_006727049.2 | Leptonychotes weddellii WS11-02 , LepWed1.0 scaffold00009 |
| *Lontra_canadensis* | XM_032861843.1 | XM_032861876.1 | Lontra canadensis , GSC_riverotter_1.0 scaffold1 |
| *Lynx_canadensis* | XM_030313451.2 | XM_030313453.2 | Lynx canadensis LIC74 chromosome B1, mLynCan4_v1.p |
| *Mustela_erminea* | XM_032332851.1 | XM_032332853.1 | Mustela erminea mMusErm1 chromosome 2, mMusErm1.Pri |
| *Mustela_putorius* |  | XM_004756089.2 | Mustela putorius furo ID#1420 breed Sable , MusPutFur1.0 scaffold00053 |
| *Neomonachus_schauinslandi* | XM_021702474.1 | XM_021702472.1 | Neomonachus schauinslandi , ASM220157v1 Super-Scaffold_77 |
| *Odobenus_rosmarus* |  | XM_004391596.2 | Odobenus rosmarus divergens Ivan , Oros_1.0 Scaffold1 |
| *Panthera_pardus* | XM_019464616.1 | XM_019464646.1 | Panthera pardus Maewha , PanPar1.0 scaffold3 |
| *Panthera_tigris* | XM_015541865.1 | XM_007074813.2 | Panthera tigris altaica TaeGuk , PanTig1.0 scaffold129 |
| *Phoca_vitulina* | XM_032400793.1 | XM_032400795.1 | Phoca vitulina PV1807 , GSC_HSeal_1.0 scaffold3 |
| *Puma_concolor* | XM_025921393.1 | XM_025921394.1 | Puma concolor SC36_Marlon , PumCon1.0 scaffold_2176 |
| *Suricata_suricatta* |  | XM_029943270.1 | Suricata suricatta VVHF042 chromosome 1, meerkat_22Aug2017_6uvM2_HiC |
| *Ursus_arctos_horribilis* | XM_026509745.1 | XM_026509746.1 | Ursus arctos horribilis, ASM358476v1 scaffold27 |
| *Ursus_maritimus* | XM_008707132.1 |  | Ursus maritimus Baiyulong , UrsMar_1.0 scaffold62 |
| *Vulpes_vulpes* | XM_025998106.1 | XM_025998103.1 | Vulpes vulpes strain TameXAggressive cross , VulVul2.2 scaffold28 |
| *Zalophus_californianus* | XM_027597399.1 | XM_027597401.1 | Zalophus californianus , zalCal2.2 UZVU01000015.1 |
| **Primates** |  |  |  |
| *Aotus_nancymaae* |  | XM_012437683.2 | Aotus nancymaae 86115 , Anan_2.0 Scaffold240.115 |
| *Callithrix_jacchus* |  | XM_008992998.2 |  |
| *Cebus_capucinus* | XM_017545100.1 | XM_017545102.1 | Cebus capucinus imitator Cc_AM_T3 , Cebus_imitator-1.0 Scaffold177 |
| *Cercocebus_atys* | XM_012040110.1 | XM_012040114.1 | Cercocebus atys FAK , Caty_1.0 Scaffold48 |
| *Chlorocebus_sabaeus* | XM_007999226.1 | XM_007999221.1 | Chlorocebus sabaeus 1994-021 chromosome 7, Chlorocebus_sabeus 1.1 |
| *Colobus_angolensis* | XM_011932393.1 |  | Colobus angolensis palliatus OR3802 , Cang.pa_1.0 Scaffold615 |
| *Gorilla_gorilla* | XM_019025377.2 | XM_019025378.2 | Gorilla gorilla gorilla Kamilah chromosome 4, Kamilah_GGO_v0 |
| *Homo_sapiens* | BC140716.1 | AF336798.1 | Homo sapiens chromosome 4, GRCh38.p13 Primary Assembly |
| *Hylobates_moloch* | XM_032142359.1 | XM_032166504.1 | Hylobates moloch HMO894 , HMol_V2 SCAF_00002 |
| *Macaca_fascicularis* | XM_005555394.2 |  | Macaca fascicularis chromosome 5, Macaca_fascicularis_5.0 |
| *Macaca_mulatta* | XM_015138772.2 | XM_028848618.1 | Macaca mulatta AG07107 chromosome 5, Mmul_10 |
| *Macaca_nemestrina* |  | XM_011737721.1 | Macaca nemestrina M95218 , Mnem_1.0 Scaffold77 |
| *Mandrillus_leucophaeus* |  | XM_011990765.1 | Mandrillus leucophaeus KB7577 , Mleu.le_1.0 Scaffold381 |
| *Nomascus_leucogenys* | XM_012499355.2 | XM_003265890.4 | Nomascus leucogenys Asia chromosome 9, Asia_NLE_v1 |
| *Pan_paniscus* | XM_008963661.2 |  |  |
| *Pan_troglodytes* | XM_024356171.1 | XM_001160851.4 | Pan troglodytes chromosome 4, Clint_PTRv2 |
| *Papio_anubis* | XM_021938431.2 | XM_031664602.1 | Papio anubis 15944 chromosome 3, Panubis1.0 |
| *Piliocolobus_tephrosceles* | XM_023195421.2 | XM_026448058.2 | Piliocolobus tephrosceles RC106 chromosome 3, ASM277652v3 |
| *Pongo_abelii* | XM_024246044.1 | XM_024246004.1 | Pongo abelii Susie chromosome 4, Susie_PABv2 |
| *Rhinopithecus_roxellana* | XM_010356902.2 |  | Rhinopithecus roxellana Shanxi Qingling chromosome 2, ASM756505v1 |
| *Saimiri_boliviensis* |  | XM_003924004.2 | Saimiri boliviensis boliviensis 3227 , SaiBol1.0 scaffold00010 |
| *Sapajus_apella* | XM_032239641.1 | XM_032239643.1 | Sapajus apella SASKATOON/1434 , GSC_monkey_1.0 scaffold45 |
| *Theropithecus_gelada* | XM_025386515.1 | XM_025386516.1 | Theropithecus gelada Dixy chromosome 5, Tgel_1.0 |
| *Trachypithecus_francoisi* | XM_033214793.1 | XM_033214782.1 | Trachypithecus francoisi TF-2019V2 , Tfra_2.0 Lachesis_group1 |
| *Carlito_syrichta* |  |  | Carlito syrichta Samal-C Ts95f , Tarsius_syrichta-2.0.1 Scaffold303036 |
| **Rodents** |  |  |  |
| *Arvicanthis_niloticus* |  |  | Arvicanthis niloticus mArvNil1 chromosome 4, mArvNil1.pat.X |
| *Castor_canadensis* | XM_020174492.1 | XM_020154239.1 | Castor canadensis Ward , C.can genome v1.0 scaffold_2012 |
| *Cavia_porcellus* |  |  | Cavia porcellus strain inbred line 2N , Cavpor3.0 supercont2_48 |
| *Chinchilla_lanigera* | XM_013502750.1 |  | Chinchilla lanigera Chin_1 , ChiLan1.0 scaffold00073 |
| *Cricetulus_griseus* |  | XM_027429726.1 | Cricetulus griseus , CriGri_1.0 scaffold3 |
| *Dipodomys_ordii* | XM_013029717.1 |  | Dipodomys ordii 6190 , Dord_2.0 Scaffold123 |
| *Fukomys_damarensis* |  |  | Fukomys damarensis Sample0158 , DMR_v1.0_HiC HiC_scaffold_32 |
| *Grammomys_surdaster* |  | XM_028789397.1 | Grammomys surdaster TR1022 , NIH_TR_1.0 125832 |
| *Heterocephalus_glaber* | XM_021261062.1 |  | Heterocephalus glaber NMR 29 , HetGla_female_1.0 scaffold00028 |
| *Ictidomys_tridecemlineatus* | XM_021734712.1 |  | Ictidomys tridecemlineatus #75 , SpeTri2.0 scaffold00217 |
| *Jaculus_jaculus* | XM_004665396.1 |  | Jaculus jaculus JJ0015 , JacJac1.0 scaffold00079 |
| *Marmota_flaviventris* | XM_027926562.1 | XM_027926564.1 | Marmota flaviventris SJ_83 , GSC_YBM_2.0 scaffold4 |
| *Marmota_marmota* | XM_015484546.1 | XM_015484547.1 | Marmota marmota marmota , marMar2.1 |
| *Mastomys_coucha* |  | XM_031382026.1 | Mastomys coucha ucsf_1 , UCSF_Mcou_1 pScaffold20 |
| *Meriones_unguiculatus* |  |  | Meriones unguiculatus strain 243 , MunDraft-v1.0 scaffold2614 |
| *Mesocricetus_auratus* |  | XM_013117723.2 | Mesocricetus auratus , MesAur1.0 scaffold00059 |
| *Microtus_ochrogaster* |  | XM_005360886.3 | Microtus ochrogaster Prairie Vole_2 linkage group LG3, MicOch1.0 |
| *Mus_caroli* |  | XM_021164082.2 | Mus caroli chromosome 6, CAROLI_EIJ_v1.1 |
| *Mus_musculus* |  | NM_025992.2 | Mus musculus strain C57BL/6J chromosome 6, GRCm38.p6 C57BL/6J |
| *Mus_pahari* |  | XM_021190750.2 | Mus pahari chromosome 2, PAHARI_EIJ_v1.1 |
| *Nannospalax_galili* | XM_008844671.3 | XM_029569086.1 | Nannospalax galili Female #2095 , S.galili_v1.0 scaffold441 |
| *Octodon_degus* | XM_004646584.1 |  | Octodon degus 3935 , OctDeg1.0 scaffold00272 |
| *Peromyscus_leucopus* |  | XM_028895246.1 | Peromyscus maniculatus bairdii , Pman_1.0 Scaffold561 |
| *Rattus_norvegicus* |  | XM_008762963.2 | Rattus norvegicus strain mixed chromosome 4, Rnor_6.0 |
| *Rattus_rattus* |  | XM_032906418.1 | Rattus rattus New Zealand chromosome 6, Rrattus_CSIRO_v1 |
| *Urocitellus_parryii* | XM_026405200.1 | XM_026405199.1 | Urocitellus parryii AGS 11-09-20 , ASM342692v1 scaffold_313 |

**Supplementary Table 1.** Information on publicly available datasets analyzed in this study. Accession numbers are available in NCBI (https://www.ncbi.nlm.nih.gov/).
